# Association of *KRTAP24-1* Gene Polymorphisms with Wool Traits in Tibetan Sheep (*Ovis aries*)

**DOI:** 10.3390/ani16132111

**Published:** 2026-07-07

**Authors:** Hongjie Zhao, Shike Ma, Wu Sun, Yujie Lu, Xiayang Jin

**Affiliations:** 1 Academy of Animal and Veterinary Sciences, Qinghai University, Xining 810016, China; 18298305354@163.com (H.Z.); mashi2019990019@163.com (S.M.); sunwufrank@163.com (W.S.); 2 Gansu Provincial General Station of Animal Husbandry Technology Extension, Lanzhou 730030, China; luyujie1113@163.com; 3 Key Laboratory of Animal Genetics and Breeding on Tibetan Plateau, Ministry of Agriculture and Rural Affairs, Lanzhou 730050, China

**Keywords:** Tibetan sheep, *KRTAP24-1*, polymorphism, haplotype, wool traits, tissue expression, protein structure

## Abstract

*KRTAP24-1* belongs to the high-sulphur KAP family and has been associated with cashmere fibre diameter in goats, but its role in ovine wool traits remains unclear. This study assessed *KRTAP24-1* tissue expression by RT-qPCR and investigated genetic variation and associations with wool traits in 277 Tibetan sheep. Polymorphisms in the coding region were identified by PCR amplification and Sanger sequencing, and genotyping was performed using PARMS. A linear mixed model (LMM) incorporating a genomic relationship matrix (GRM) was used to evaluate associations between SNPs, haplotypes, and 12 wool traits. Bioinformatic analyses were restricted to the five haplotypes observed in the study population and were used as preliminary in silico assessments. Three missense SNPs were identified: c.191C>T (p.L64P), c.527G>A (p.G176D), and c.656C>T (p.A219V). The c.191C>T variant was associated with mean fibre length (MFL), single fibre tenacity (SFT), and scoured yield (SY), whereas c.656C>T was associated with lock length (LL) and clean fleece yield (CFY). Several haplotype combinations were also associated with LL, elongation at break (EB), and CFY. *KRTAP24-1* showed high expression in skin. The observed haplotypes showed only minor differences in predicted mRNA secondary structure and mainly local changes in predicted protein features. These findings suggest that *KRTAP24-1* may provide a preliminary basis for marker-assisted selection in Tibetan sheep breeding, but its functional role requires further validation in independent populations and experimental systems.

## Simple Summary

This study focused on the keratin-associated protein (*KRTAP24-1*) and examined its genetic variation and associations with wool traits in Tibetan sheep. We identified three missense mutations and found that several genotypes and haplotype combinations were associated with wool length, clean fleece yield, and fibre mechanical traits. Bioinformatic analyses based on the observed haplotypes provided preliminary in silico evidence that these variants may be associated with minor local changes in mRNA secondary structure and protein features, but these predictions require further experimental validation.

## 1. Introduction

Tibetan sheep are an indigenous breed unique to the Qinghai–Tibet Plateau. Following long-term natural selection, they have developed remarkable ecological adaptation to extreme cold, hypoxia, and intense ultraviolet radiation [1,2]. Their fleece is not only a key physiological barrier against severe cold, but also an important source of income for local herders [3]. Owing to its long fibres, good elasticity, and high tensile strength, Tibetan sheep wool is commonly used to produce Tibetan carpets and handmade textiles [4]. However, wool traits such as fineness, length, and mechanical strength are typical quantitative traits controlled by multiple genes with small effects and are regulated by complex genetic networks [5]. Therefore, elucidating the underlying molecular genetic mechanisms is essential for improving wool quality.

Keratin-associated proteins (KAPs), together with keratin intermediate filaments (KIFs), are key structural components of the wool fibre matrix, with KAPs accounting for approximately 30–40% of the fibre dry weight [6]. Through dense disulphide cross-linking with KIFs, KAPs form a stable and rigid network structure, thereby contributing to physical fibre traits such as fineness and strength [7]. Among the 26 identified KRTAP subfamilies, high glycine–tyrosine (HGT) proteins play an important role in cortical assembly during the early stages of hair follicle development [8]. The *KRTAP24-1* gene is one of the core members of the KRTAP family. Although Zhou et al. [9] initially identified four sequence variants of *KRTAP24-1* in New Zealand Romney-cross sheep using SLCP technology, their study was limited to gene identification and sequence analysis, and did not establish associations between genotypes and wool phenotypic traits. In goats, Wang et al. [10] demonstrated that SNP variation in this gene significantly affects cashmere fibre diameter. Given the high conservation of the KRTAP family during ruminant evolution, we hypothesised that *KRTAP24-1* may also be a key candidate gene regulating the physical properties of ovine wool. To date, no study has investigated the association between polymorphisms in this gene and wool phenotypes in sheep, and information on plateau-adapted Tibetan sheep remains unavailable. Thus, whether *KRTAP24-1* polymorphisms regulate wool fibre fineness, length, and related traits lacks experimental evidence.

Accordingly, this study examined the tissue expression characteristics of *KRTAP24-1* in Tibetan sheep. We next screened the coding region for genetic variation using Sanger sequencing and PARMS genotyping, and analysed the association of genotypes with wool traits through a linear mixed model (LMM). In addition, we also used bioinformatic tools to predict the functional consequences of missense mutations from microscopic perspectives, including mRNA secondary structure stability and changes in protein monomer energy. This study aimed to reveal genetic variation in *KRTAP24-1* in plateau Tibetan sheep and to provide preliminary candidate molecular markers for the genetic improvement of wool traits in Tibetan sheep.

## 2. Materials and Methods

### 2.1. Animals and Feeding Conditions

The Animal Ethics Committee of Qinghai University reviewed and approved this study (approval No. 2026-QHMKY-009). We sampled 277 healthy Tibetan sheep (238 females, 39 males; aged 2–4 years) from the Modern Ecological Animal Husbandry Technology Demonstration Park in Haiyan County, Haibei Prefecture, Qinghai Province (approximately 3400 m above sea level). All sheep were managed under identical conditions with natural light, free access to water, grazing on native pasture during the day, and housing at night. The animals were collected in two batches: 199 on 9 January 2026 and 78 on 22 January 2026. Pedigree relationships among individuals are incompletely recorded; population genetic structure was corrected via a genomic relationship matrix (GRM, Supplementary File S1) to exclude false positive associations potentially caused by genetic relatedness among individuals [11]. The GRM was constructed using VanRaden’s method [12] based on whole-genome sequencing data (average 10× depth) from all 277 samples. WGS was implemented following the GATK best practices pipeline as described by McKenna et al. [13].

### 2.2. Collection of Blood, Tissue, and Wool Sample

We collected 5 mL of jugular venous blood into EDTA-coated tubes, mixed gently, transported at 4 °C to the laboratory, and stored at −20 °C for genomic DNA extraction;

Approximately 30 g of wool was clipped from the left flank approximately 10 cm behind the scapula, sealed in a plastic bag, and stored for subsequent trait measurement.

Three animals were randomly selected for tissue sampling. After slaughter, skin (with hair follicles), heart, liver, spleen, lung, kidney, muscle, ovary, large intestine, small intestine, subcutaneous fat, mammary gland, and rumen tissue were collected, immediately snap-frozen in liquid nitrogen, and stored at −80 °C for RNA extraction and gene expression analysis.

### 2.3. Wool Traits Analysis

Staple length (SL) and lock length (LL)was measured in vivo with a steel ruler at approximately 10 cm behind the left scapula, concurrently with blood collection. For SL, we parted the outer coarse fibres and measured the natural distance from the skin end to the tip of the inner staple comprising fine wool and heterotypic fibres. For LL, we selected typical locks formed by outer coarse (hair) fibres and measured the vertical hanging distance from the skin surface to the lock tip while the lock hung naturally. At least three natural staples or locks were measured per animal for each trait, and the mean was used as the individual phenotypic value. Mean fibre length (MFL), coefficient of variation of fibre length (CVFL), mean fibre diameter (MFD), fibre diameter standard deviation (FDSD), coefficient of variation of fibre diameter (CVFD), single fibre breaking force (SFBF), elongation at break (EB), single fibre tenacity (SFT), scoured yield (SY), and clean fleece yield (CFY). Detailed measurement methods and instrument parameters for each trait are provided in Supplementary File S2.

### 2.4. RNA Extraction and RT-qPCR Analysis

Total RNA was extracted using TRIzol reagent [14]. RNA concentration and purity were measured with a NanoDrop™ 2000 spectrophotometer (Thermo Fisher Scientific, Waltham, MA, USA), and RNA integrity was assessed on an Agilent 2100 Bioanalyzer (Agilent Technologies, Santa Clara, CA, USA). The extracted RNA met the following criteria: OD_260/280_ ratio between 1.8 and 2.0, OD_260/230_ ratio above 2.0, and RIN value between 7.5 and 8.5.

Primers targeting the sheep *KRTAP24-1* gene (GenBank accession No. XM_027957911.2) were designed with Primer Premier 5.0 and verified using NCBI Primer-BLAST (https://blast.ncbi.nlm.nih.gov, accessed on 15 April 2026). The primers were synthesised by Yangling Tianrun Aoke Biotechnology Co., Ltd. (Yangling, China). *GAPDH* was used as the reference gene [15,16]. Primer sequences, reaction mixtures, and thermal cycling conditions are listed in Supplementary Tables S1 and S3. Reverse transcription was performed using the PrimeScript™ RT Reagent Kit with gDNA Eraser (RR047A, Takara Biomedical Technology (Beijing) Co., Ltd., Beijing, China). RT-qPCR was conducted using the SYBR Green method (Talent qPCR PreMix (SYBR Green), Tiangen Biochemical Technology (Beijing) Co., Ltd., Beijing, China) on an Applied Biosystems QuantStudio 6 Flex real-time PCR system (Thermo Fisher Scientific, Waltham, MA, USA). Relative expression levels of *KRTAP24-1* across tissues were calculated using the 2^−ΔΔCt^ method with heart tissue as the calibrator. Samples that failed to reach the quantification threshold or lacked reliable amplification signals were conservatively assigned a Ct value of 40 for relative expression calculation. Three biological replicates were analysed per tissue. Differences in expression among tissues were assessed by ANOVA followed by Tukey’s HSD post hoc test, with *p* < 0.05 considered statistically significant. Results were visualised using the ggplot2 (version 4.0.3) and multcompView (version 0.1-10) packages in R (version 4.4.2).

### 2.5. DNA Extraction, PCR Amplification, and Genotyping of KRTAP24-1 Polymorphisms

Genomic DNA was extracted from thawed blood samples using the Animal Blood Genomic DNA Extraction Kit (DP304, Tiangen Biochemical Technology (Beijing) Co., Ltd., Beijing, China). DNA concentration and purity were measured with a NanoDrop™ 2000 (Thermo Fisher Scientific, Waltham, MA, USA). Samples with a concentration above 20 ng/µL and an OD_260/280_ ratio between 1.7 and 1.9 were considered acceptable.

PCR primers were designed based on the sheep *KRTAP24-1* reference sequence (GenBank accession No. XM_027957911.2). Primer sequences are provided in Supplementary Table S1, and the primers were synthesised by Yangling Tianrun Aoke Biotechnology Co., Ltd. (Yangling, China). The exon region of *KRTAP24-1* was amplified in 16 randomly selected individuals from the cohort of 277. PCR reaction mixtures and thermal cycling conditions are listed in Supplementary Table S2. Amplification products were examined on a 1.5% agarose gel, and samples producing a single, clear band were selected for sequencing. Two microlitres of each PCR product was sent to Yangling Tianrun Aoke Biotechnology Co., Ltd. (Yangling, China) for bidirectional Sanger sequencing using the forward and reverse PCR primers. Only chromatograms with Phred quality scores ≥30 across the target region, clear and well-resolved single peaks at each nucleotide position, and 100% concordance between forward and reverse reads were accepted for downstream analysis. Sequence alignment was performed using DNAMAN software (version 8.2.2). After identifying the variant sites, all 277 Tibetan sheep DNA samples were genotyped using the penta-primer amplification refractory mutation system (PARMS) (Jingtai Biotechnology Co., Ltd., Wuhan, China). PARMS genotyping quality was assessed by including no-template controls on each plate and by requiring samples to fall within clearly separated fluorescence clusters (FAM and HEX) with call confidence > 95%. Samples clustering near the origin or outside defined clusters were excluded. The genotyping primer sequences are listed in Supplementary Table S4.

### 2.6. Genetic Diversity Analysis

Data were organised using Excel 2024. Allele frequency, genotype frequency, polymorphism information content (PIC), expected heterozygosity (He), observed heterozygosity (Ho), effective number of alleles (Ne), and Hardy–Weinberg equilibrium (HWE) tests were calculated using the genetics package (version 1.3.8.1.3) in R (version 4.4.2). Linkage disequilibrium (LD) analysis was performed using the SHEsisPlus online platform (http://analysis.bio-x.cn/) to calculate D′ and R^2^ values. Haplotype analysis was conducted using the geneHapR package (version 1.2.4) in R (version 4.4.2) [17].

### 2.7. Statistical Analysis

Based on the wool trait measurements, the association of genotypes and haplotype combination with phenotypic traits was evaluated using a linear mixed model (LMM) implemented in R (version 4.4.2). When the frequency of a genotype or haplotype combination was less than 5%, the mean and variance estimates derived from extremely small sample groups were unstable, their statistical power was insufficient to support meaningful comparisons, and they were prone to false positive associations. Such genotypes and haplotype combinations were therefore excluded from subsequent association analyses [18]. The models were as follows:

*Y_ijkl_* = *μ* + *M_i_* + *S_j_* + *A_k_* + *B_l_* + *u* + *e_ijkl_* (1)

*Y_ijkl_* = *μ* + *H_i_* + *S_j_* + *A_k_* + *B_l_* + *u* + *e_ijkl_* (2)

where *Y_ijkl_* is the phenotypic value of the trait, μ is the overall mean, *M_i_* is the genotype effect, *H_i_* is the diplotype effect, *S_j_*, *A_k_*, and *B_l_* are the effects of sex, age, and sampling batch, respectively, *u* is the random polygenic effect for each individual, and *e_ijkl_* is the random residual. Genotype, diplotype, sex, age, and sampling batch were included as fixed effects. The random polygenic effect was assumed to follow u ~ N (0, Gσ^2^u), where G is the genomic relationship matrix (GRM) constructed from whole-genome resequencing data of the 277 Tibetan sheep, and σ^2^u is the polygenic genetic variance. The residual was assumed to follow e ~ N (0, Iσ^2^e). Traits that did not satisfy normality (Shapiro–Wilk test, *p* < 0.05) were normalised using rank-based inverse normal transformation (RINT) prior to analysis. The models were fitted using the mmer function of the sommer package (version 4.4.5) in R (version 4.4.2). The significance of the main effects of genotype and diplotype was assessed using Wald χ^2^ tests. The results are presented as raw means ± standard deviations. Wald test *p*-values for each SNP and each diplotype group across the 12 wool traits were corrected for multiple testing using the Benjamini–Hochberg false discovery rate (FDR) method. Associations with FDR-adjusted *p* < 0.05 were considered statistically significant, and those with FDR-adjusted *p* < 0.01 were considered highly significant. Post hoc pairwise comparisons were performed using Tukey–Kramer HSD tests.

### 2.8. Bioinformatic Analysis

Based on the five *KRTAP24-1* haplotype sequences identified in the population, the mRNA secondary structure was predicted using the RNAfold Web Server (http://rna.tbi.univie.ac.at/cgi-bin/RNAWebSuite/RNAfold.cgi, accessed on 18 April 2026) at the default temperature of 37 °C. The physicochemical properties of the five *KRTAP24-1* haplotypes were computed using the ExPASy ProtParam tool (https://web.expasy.org/protparam/, accessed on 18 April 2026). Signal peptides and transmembrane domains were predicted with SignalP 6.0 (https://services.healthtech.dtu.dk/services/SignalP-6.0/, accessed on 18 April 2026) and DeepTMHMM 1.0 (https://services.healthtech.dtu.dk/services/DeepTMHMM-1.0/, accessed on 18 April 2026), respectively. Protein secondary structure was predicted using SOPMA (https://npsa-prabi.ibcp.fr/cgi-bin/npsa_automat.pl?page=npsa_sopma.html, accessed on 18 April 2026). The tertiary structure of KRTAP24-1 protein was modelled using SWISS-MODEL (https://swissmodel.expasy.org/) with the AlphaFold Database entry AF-G3N073-F1 (UniProt accession G3N073, a sheep KAP protein) as the template. The target-template alignment yielded a sequence identity of 90.84%, sequence similarity of 60%, and coverage of approximately 95%. Model quality was assessed as GMQE = 0.44. The predicted PDB file is provided as Supplementary File S3. Based on this predicted structure, MutationExplorer (https://mutationexplorer.vda-group.de/mutationexplorer/, accessed on 18 April 2026) was employed to introduce the three missense mutations and calculate changes in local residue energy and total protein energy, expressed in Rosetta energy units (REU), to evaluate the direction of energy change (increase or decrease) associated with each substitution. A protein–protein interaction network was constructed using the STRING database v12.0 (https://string-db.org/, accessed on 19 April 2026) at a high-confidence threshold (combined score ≥ 0.7) and visualized with Cytoscape 3.10.4 (https://cytoscape.org/, accessed on 19 April 2026).

## 3. Results

### 3.1. Tissue Expression of KRTAP24-1

**Figure 1:**
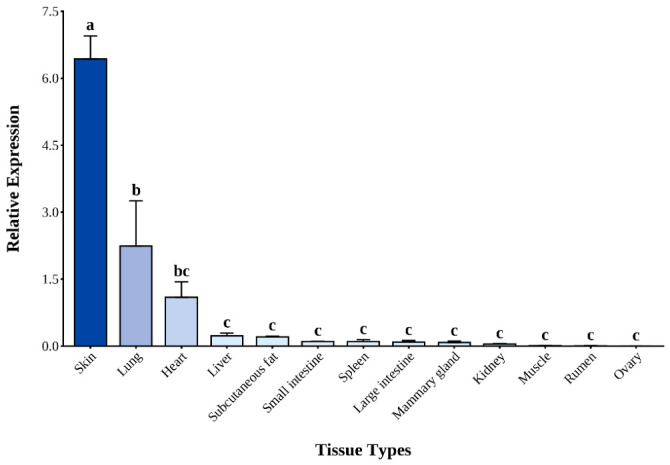
The mRNA expression level of *KRTAP24-1* gene in different tissues of Tibetan sheep. Expression was normalized to the reference gene *GAPDH* using the 2^−ΔΔCt^ method, with heart tissue as the calibrator. Tissues with undetectable amplification were assigned a Ct value of 40. Data are presented as mean ± SE (*n* = 3 biological replicates). Different lowercase letters indicate significant differences (*p* < 0.05, Tukey’s HSD).

RT-qPCR analysis revealed that *KRTAP24-1* expression in skin tissue was significantly higher than in all other tissues examined (*p* < 0.05). Detectable expression was observed in the lung and heart, whereas only minimal levels were found in the liver and subcutaneous fat (Figure 1). Expression was virtually absent in the mammary gland, muscle, rumen, small intestine, spleen, large intestine, kidney, and ovary. These findings provide a preliminary investigation into the tissue expression of *KRTAP24-1*, which requires further validation using larger sample sizes.

### 3.2. Detection and Genotyping of KRTAP24-1 Polymorphisms in Tibetan Sheep

**Figure 2:**
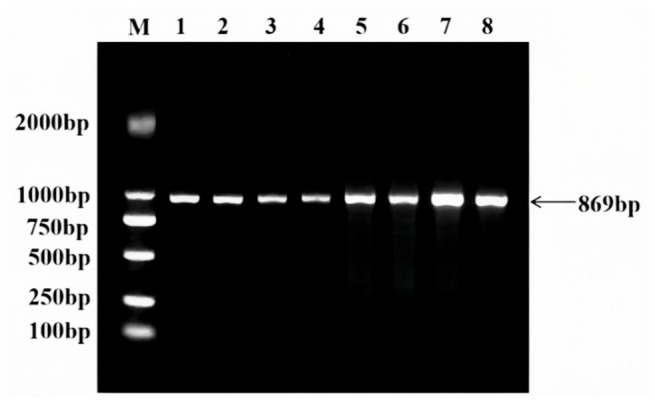
PCR amplification of the *KRTAP24-1*. Lanes 1–8: amplified target fragments (expected size: 869 bp) from representative individual Tibetan sheep samples; M: DL2000 DNA Marker.

**Figure 3:**
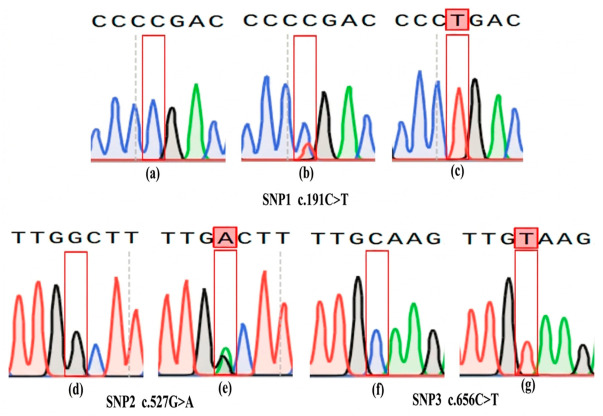
Sequencing chromatograms of three single-nucleotide polymorphisms in the *KRTAP24-1* gene based on Sanger sequencing of 16 randomly selected Tibetan sheep samples. The red blocks indicate the mutation site. (**a**) SNP1 (c.191C>T) CC genotype; (**b**) SNP1 CT genotype; (**c**) SNP1 TT genotype; (**d**) SNP2 (c.527G>A) GG genotype; (**e**) SNP2 GA genotype; (**f**) SNP3 (c.656C>T) CC genotype; (**g**) SNP3 CT genotype. The homozygous mutant genotypes of SNP2 (AA) and SNP3 (TT) were not observed among the 16 sequenced individuals but were identified by PARMS genotyping of the full population.

**Figure 4:**
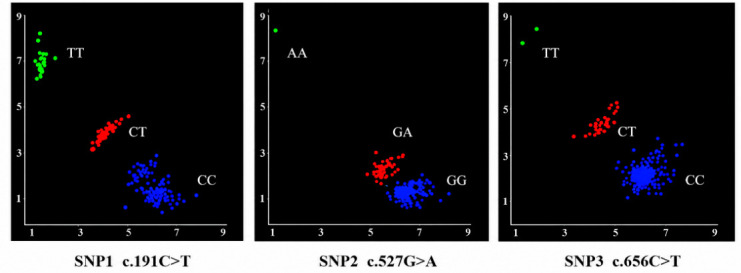
Allelic discrimination scatter plots of three single-nucleotide polymorphisms in the *KRTAP24-1* genotyped by PARMS across the full population (n = 277). Each data point represents an individual animal, with its position determined by the relative fluorescence intensity in the FAM and HEX channels. SNP1 (c.191C>T): three clusters corresponding to CC (FAM), CT (FAM + HEX), and TT (HEX) genotypes; SNP2 (c.527G>A): three clusters corresponding to GG (FAM), GA (FAM + HEX), and AA (HEX) genotypes; SNP3 (c.656C>T): three clusters corresponding to CC (FAM), CT (FAM + HEX), and TT (HEX) genotypes. Dots near the origin represent failed reactions, which were excluded from genotype assignment.

An 869 bp fragment covering the coding region of *KRTAP24-1* (GenBank accession No. XM_027957911.2) was successfully amplified. Agarose gel electrophoresis showed clear and specific amplification, indicating that the primers were suitable for subsequent analysis (Figure 2). Sanger sequencing was first performed on 16 randomly selected individuals for initial variant discovery (Figure 3). Based on the sequencing results, three single-nucleotide polymorphisms were identified: SNP1(c.191C>T), SNP2(c.527G>A) and SNP3 (c.656C>T). All three were exonic missense mutations, predicting amino acid substitutions p.L64P, p.G176D, and p.A219V, respectively (Table 1). These three variants were then genotyped across the full cohort of 277 Tibetan sheep using the PARMS (Figure 4). The genotyping call rates for SNP1, SNP2, and SNP3 were 98.6%, 97.1%, and 98.9%, respectively. The missing genotypes resulted from failed PARMS reactions in a small number of samples, which were excluded from downstream association analyses.

### 3.3. Linkage Disequilibrium and Haplotype Architecture

**Figure 5:**
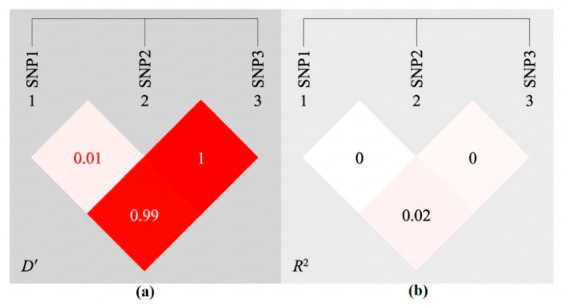
Pairwise linkage disequilibrium (LD) analysis of three SNPs in the *KRTAP24-1* gene. (**a**) shows the standardised LD coefficients (*D′*); (**b**) shows the squared correlation coefficients (*R*^2^) between each SNP pair. The *D′* values represent the degree of historical linkage, ranging from 0 (no LD) to 1.00 (complete LD). The *R*^2^ values represent the statistical correlation between alleles at each pair of loci, ranging from 0 (no correlation) to 1.00 (perfect correlation). The colour intensity of each cell reflects the magnitude of the respective LD metric, with deeper red indicating stronger LD.

LD analysis revealed different linkage patterns among the three SNPs in the *KRTAP24-1* gene (Figure 5). Both the *D′* and *R*^2^ values for SNP1–SNP2 were close to zero (*D′* = 0.01, *R*^2^ = 0.00), indicating that these two loci were in near-complete linkage equilibrium in this population, with allelic combinations approaching randomness. In contrast, SNP2–SNP3 and SNP1–SNP3 exhibited relatively high *D′* values (1.00 and 0.99, respectively), suggesting the presence of strong historical linkage between these loci. However, the low *R*^2^ values indicated weak allelic correlations, implying limited predictive power of one locus for the other. Haplotype analysis results are shown in Table 2. Five haplotypes were identified in the population, among which Haplotype 1 (CGC) had the highest frequency (59.13%) and Haplotype 5 (TAC) had the lowest (3.23%).

### 3.4. Analysis of Population Genetic Parameters

Population genetic diversity analysis of the three SNPs in the *KRTAP24-1* gene showed that at the c.191C>T locus, three genotypes were detected—CC, CT, and TT—with CC being the predominant genotype and C the dominant allele. At the c.527G>A locus, three genotypes were detected, with GG being predominant, AA being rare, and G being the dominant allele. At the c.656C>T locus, three genotypes were also detected, with CC being predominant, TT being rare, and C being the dominant allele. The polymorphism information content (*PIC*) values were 0.3053 for c.191C>T, indicating moderate polymorphism (0.25 < *PIC* < 0.50), and 0.1808 and 0.1289 for c.527G>A and c.656C>T, respectively, indicating low polymorphism (*PIC* < 0.25). The expected heterozygosity (*He*) values were 0.3759, 0.2011, and 0.1384; the observed heterozygosity (*Ho*) values were 0.2821, 0.2193, and 0.1350; and the effective numbers of alleles (*Ne*) were 1.602, 1.251, and 1.161, respectively. Hardy–Weinberg equilibrium testing showed that the c.191C>T locus deviated significantly from equilibrium (*p* < 0.001), whereas the c.527G>A and c.656C>T loci were both in equilibrium (*p* > 0.05) (Table 3).

### 3.5. Association Analysis of KRTAP24-1 Variants and Haplotype Combinations with Ovine Wool Traits

Because the AA genotype (*n* = 1) of SNP2 and the TT genotype (*n* = 2) of SNP3 had genotype frequencies below 5%, provides insufficient statistical power for meaningful comparison and is highly sensitive to outliers; thus, they were excluded from the association analysis between *KRTAP24-1* genotypes and wool traits, and only the remaining individuals were included in the statistical analysis (Table 4). For the SNP1 (c.191C>T) locus, the mean fibre length (MFL) of TT genotype individuals was significantly higher than that of the other two genotypes (*p* < 0.05), while the single fibre tensile strength (SFT) was highly significantly lower than the other two genotypes (*p* < 0.01), and the scoured yield (SY) was significantly lower than the other two genotypes (*p* < 0.05). For the SNP2 (c.527G>A) locus, no significant differences were detected between genotypes and wool phenotypic traits (*p* > 0.05). For the SNP3 (c.656C>T) locus, CC genotype individuals exhibited significantly longer lock length (LL) than CT genotype individuals (*p* < 0.05), and a highly significantly higher clean fleece yield (CFY) than CT genotype individuals (*p* < 0.01).

The associations between haplotype combinations and wool traits were further examined (Table 5). Samples with haplotype combination frequencies below 5.0% were excluded from the analysis, leaving six haplotype combinations in the population: H1H1, H1H2, H1H3, H1H4, H2H5, and H2H2. The complete results are provided in Supplementary File S5. Compared with the other haplotype combinations, Tibetan sheep carrying H2H5 had significantly greater lock length (LL) (*p* < 0.05). Individuals with H1H1 had significantly higher elongation at break (EB) (*p* < 0.05), whereas those with H2H2 had significantly higher clean fleece yield (CFY) than sheep with the other haplotype combinations (*p* < 0.05).

### 3.6. Bioinformatic and Structural Analysis of KRTAP24-1

**Figure 6:**
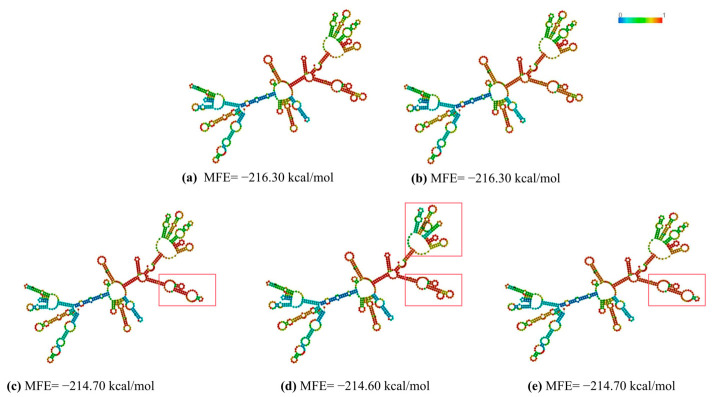
Predicted mRNA secondary structures of five *KRTAP24-1* haplotypes identified by RNAfold (temperature: 37 °C). (**a**) H1; (**b**) H2; (**c**) H3; (**d**) H4; (**e**) H5. Local structural variations between haplotypes are highlighted by red boxes.

Based on the sequences of the five observed haplotypes (H1–H5), the mRNA secondary structures of KRTAP24-1 were predicted using RNAfold (Figure 6). All five haplotypes formed complex stem–loop structures with highly similar overall folding patterns. The minimum free energy (MFE) of H1 and H2 was −216.30 kcal/mol, while those of H3, H4, and H5 were −214.70, −214.60, and −214.70 kcal/mol, respectively. Compared with H1, the MFE of H3, H4, and H5 increased by 1.60, 1.70, and 1.60 kcal/mol, respectively. Despite the modest magnitude of these MFE differences, subtle local conformational changes were observed in specific stem–loop regions of H3, H4, and H5.

KRTAP24-1 encodes a protein of 252 amino acid residues, predominantly rich in Ser, Cys, Gly, and Leu. The amino acid compositions of the five haplotype-encoded proteins are highly similar, differing only in single-residue substitutions at the three missense mutation sites.

ExPASy ProtParam predictions (Table 6) showed that the five haplotypes were all composed of five elements: C, H, O, N, and S. The number of positively charged residues (Arg + Lys) was 21 in all haplotypes, while the negatively charged residues (Asp + Glu) increased from 10 to 11 in H3 and H5. The molecular weight ranged from 27,860.24 Da to 27,934.32 Da. The theoretical isoelectric points (pI) of H3 and H5 were 8.77, lower than those of the remaining three haplotypes (8.84). The GRAVY values ranged from −0.473 to −0.429. H2 and H5 exhibited the lowest aliphatic index (49.92), whereas H4 showed the highest (52.22). The instability index of all five variants exceeded the threshold of 40 (58.51–59.57).

**Figure 7:**
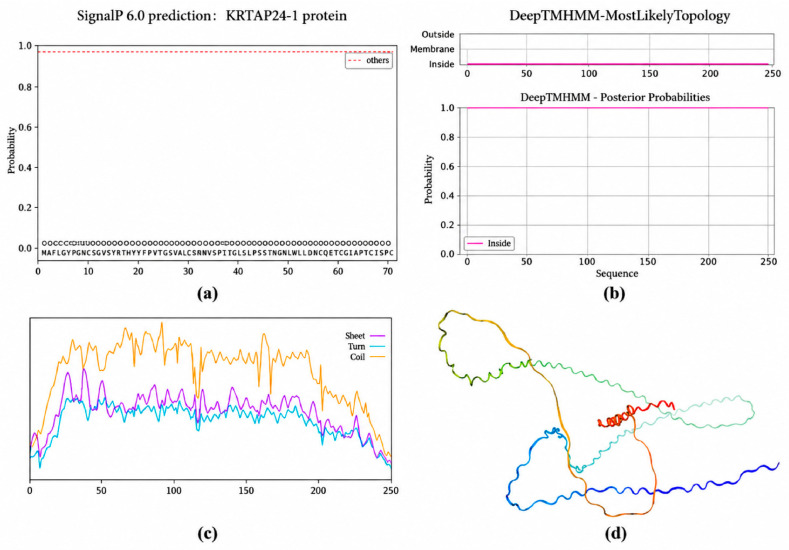
Predictions of KRTAP24-1 protein structure and properties: (**a**) Signal peptide prediction in Tibetan sheep. (**b**) Transmembrane helix prediction. (**c**) Secondary Structure Prediction of KRTAP24-1. (**d**) Predicted Tertiary Structure of the KRTAP24-1 Protein.

SignalP 6.0 prediction revealed that KRTAP24-1 contains no signal peptide (Figure 7a), indicating it is a non-secretory protein. DeepTMHMM analysis showed that KRTAP24-1 lacks transmembrane helices (Figure 7b), with a 100% posterior probability of the entire sequence being localized to the cytoplasm, further confirming its cytoplasmic localization.

SOPMA and SWISS-MODEL predictions (Figure 7c,d) demonstrated that KRTAP24-1 exhibits typical intrinsically disordered features, with its secondary structure dominated by random coils. The probability profile indicated that the central region (approximately residues 30–220) showed a substantially higher probability of random coil conformation compared to other secondary structures, whereas α-helical structures were essentially absent. All three missense mutation sites were located within flexible loop regions and did not substantially perturb the overall folding. The predicted tertiary structure also exhibited a predominantly random coil architecture.

**Figure 8:**
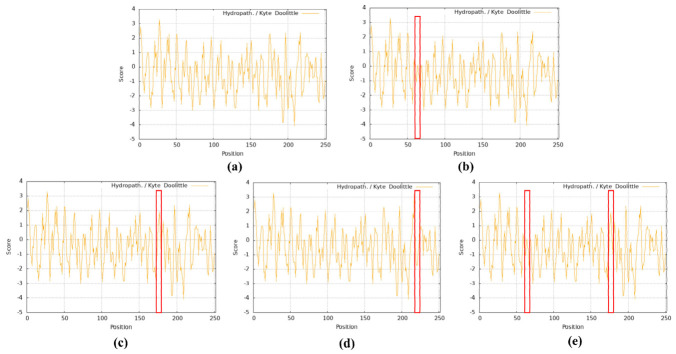
(**a**) H1 hydrophilic and hydrophobic prediction; (**b**) H2 hydrophilic and hydrophobic prediction; (**c**) H3 hydrophilic and hydrophobic prediction; (**d**) H4 hydrophilic and hydrophobic prediction; (**e**) H5 hydrophilic and hydrophobic prediction. The red frames indicate the mutation-related regions.

The Kyte–Doolittle algorithm was used to analyze the hydrophobicity profiles of the five KRTAP24-1 haplotypes (Figure 8). H1 served as the reference sequence, showing alternating hydrophobic and hydrophilic regions (Figure 8a). Compared with H1, the Leu→Pro substitution in H2 decreased the hydrophobicity score from +1.867 to +0.067 (Δ = −1.800) (Figure 8b); the Gly→Asp substitution in H3 decreased the score from +2.300 to +1.267 (Δ = −1.033) (Figure 8c); and the Ala→Val substitution in H4 increased the score from −2.067 to −1.267 (Δ = +0.800) (Figure 8d). H5 carried both the Leu→Pro and Gly→Asp substitutions, resulting in reduced hydrophobicity in the corresponding regions (Figure 8e). Overall, the hydrophobicity profiles of the five haplotypes were highly similar, with the missense mutations primarily causing local changes in hydrophobicity without markedly altering the global distribution.

**Figure 9:**
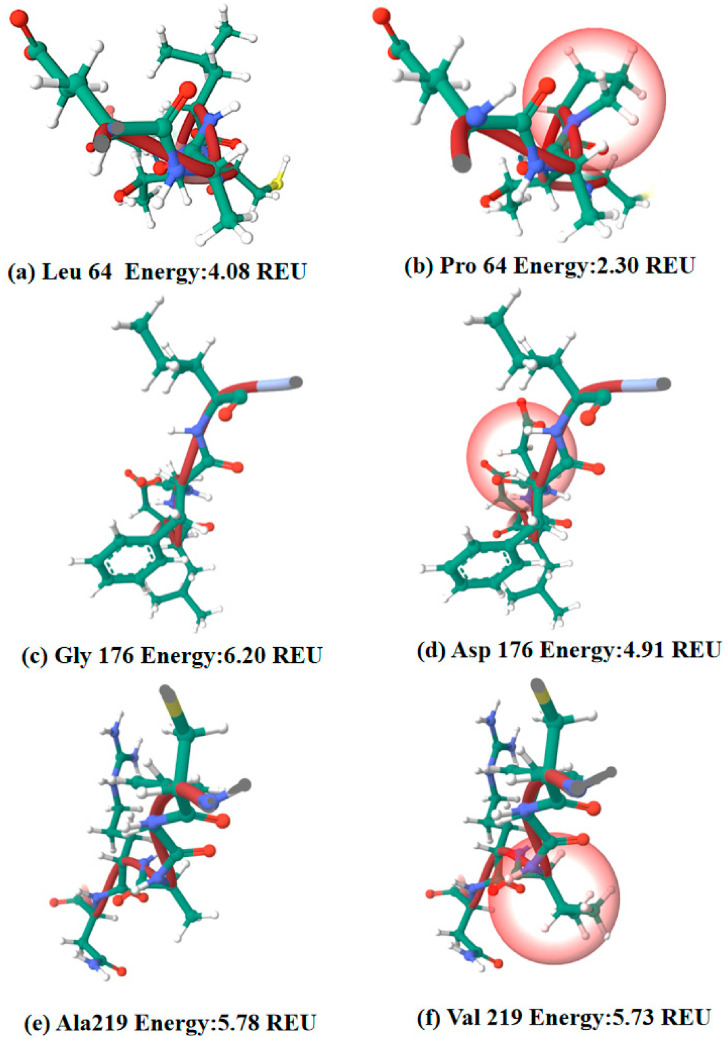
Local structural energy analysis of amino acid substitutions in KRTAP24-1 protein. (**a**) Leu64; (**b**) Pro64; (**c**) Gly176; (**d**) Asp176; (**e**) Ala219; (**f**) Val219. Energy values are shown in Rosetta energy units (REU). The highlighted regions indicate local structural changes around the substituted residues.

Local structural energy analysis of the three missense mutation sites in KRTAP24-1 was performed using Mutation Explorer (Figure 9). The results showed that the local energy values at the mutation sites changed from 4.08, 6.20, and 5.78 REU to 2.30, 4.91, and 5.73 REU for p.L64P, p.G176D, and p.A219V, respectively, corresponding to energy changes of −1.78, −1.29, and −0.05 REU. Among these, the local energy changes at p.L64P and p.G176D were relatively pronounced, suggesting that these two amino acid substitutions may induce alterations in the local energetic environment and side-chain spatial conformation near the mutation sites. The energy change at p.A219V was minimal before and after substitution, indicating that this residue replacement may have a weaker impact on the local structural environment. Structural visualization revealed local side-chain rearrangement or spatial conformational adjustments around some of the mutation sites; however, no further inference regarding the effect on overall protein stability was made.

In summary, the three missense mutations may exert differential effects on the local structural environment of KRTAP24-1, with p.L64P and p.G176D showing relatively pronounced local conformational changes, whereas the impact of p.A219V was comparatively minor.

**Figure 10:**
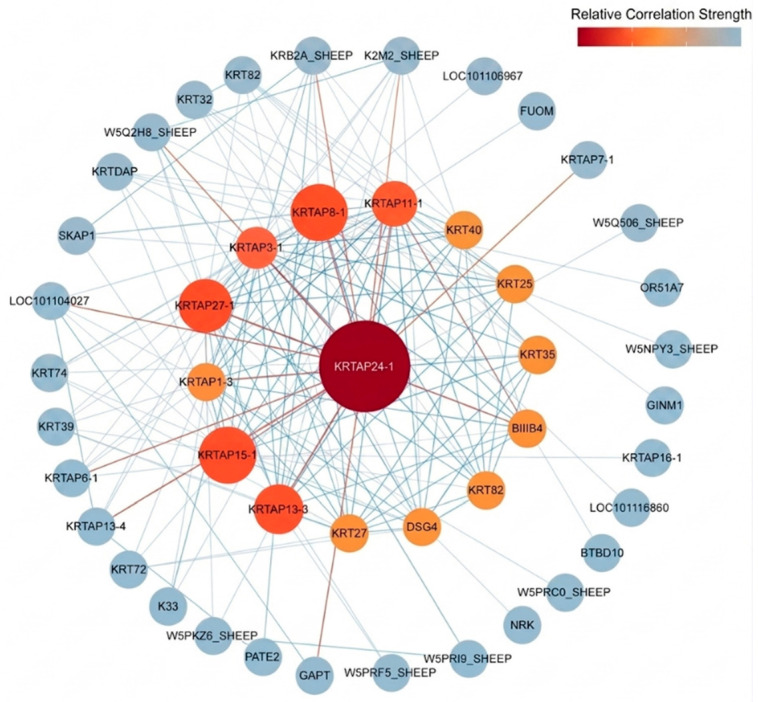
PPI Prediction Analysis of KRTAP24-1 Protein (Confidence Score > 0.7).

To further investigate the molecular mechanism of *KRTAP24-1* in wool fibre formation, a protein–protein interaction network for ovine KRTAP24-1 protein was constructed using the STRING database (combined score ≥ 0.7) (Figure 10). The results showed that KRTAP24-1 has predicted functional associations with multiple KAP family members known to be involved in wool trait regulation, including KRTAP13-3, KRTAP15-1, KRTAP27-1, KRTAP8-1, and KRTAP3-1. The evidence supporting these interactions is primarily derived from text mining and co-expression data, with partial contributions from curated databases and experimental determination, based on mammalian ortholog evidence.

## 4. Discussion

Keratins and keratin-associated proteins (KAPs) are the major structural components of wool fibres and have been widely studied in ruminants [19]. Previous studies have shown that KRTAP members differ in their expression patterns and genetic variation among goats, sheep, and different breeds, leading to variable effects on wool and cashmere traits [8,20]. This variation not only reflects functional divergence of KRTAP genes during evolution, but also suggests that their regulation of fibre traits may be species-specific and dependent on breed background. By integrating association analysis, mRNA and protein structure prediction, and protein–protein interaction network analysis, this study established a multidimensional line of evidence linking sequence variation to predicted molecular structural changes and phenotypic effects.

### 4.1. Tissue Expression Analysis of KRTAP24-1

Tissue expression profiling showed that *KRTAP24-1* was highly and specifically expressed in Tibetan sheep skin containing hair follicles. Most reported KRTAP family genes show strong skin- and hair follicle-specific expression in sheep and goats, including caprine *KRTAP6-3* [21], *KRTAP27-1* [22], and *KRTAP20-2* [23], as well as ovine *KRTAP3-3* [24] and members of the *KRTAP6* family [25]. The high expression of *KRTAP24-1* in skin suggests that it may play an important regulatory role in hair follicle development and wool formation. However, some studies have shown that the expression of caprine *KRTAP11-1* and ovine *KRTAP2-1* is not restricted to skin, as transcripts can also be detected in liver, heart, and lung tissues [26,27]. In the present study, *KRTAP24-1* was also expressed in several Tibetan sheep tissues, suggesting that it may have functions beyond the regulation of wool fibre development, although these preliminary expression trends require further investigation.

### 4.2. Population Genetic Characteristics of KRTAP24-1 in Tibetan Sheep

The three SNPs identified in this study were all missense mutations, resulting in the amino acid substitutions p.L64P, p.G176D, and p.A219V. Population genetic analysis showed that SNP1 (c.191C>T) deviated highly significantly from Hardy–Weinberg equilibrium and exhibited a marked heterozygote deficit (observed CT = 77; expected CT ≈ 103), whereas the other two loci remained in equilibrium. This deviation may be attributable to several factors. One possible explanation is population substructure, namely the Wahlund effect, arising from the extensive management system of the sampled flock, in which breeding rams may be shared among different grazing subgroups. Similar heterozygote deficits caused by flock subdivision have been widely reported in sheep populations [28,29]. In addition, limited population size and long-term selection pressure for wool traits may have further contributed to the departure from equilibrium at this locus [30]. The possibility of genotyping error is low, given the clear PARMS clustering and the consistency between Sanger sequencing and PARMS genotyping. Moreover, the linear mixed model used in this study incorporated a genomic relationship matrix to correct for population stratification, thereby improving the robustness of the reported association results. Similar deviations have also been reported in other sheep breeds; for example, Bai et al. [31] observed deviation from Hardy–Weinberg equilibrium for *KRTAP28-1* variants in New Zealand Romney × Southdown-cross lambs.

Polymorphism information content analysis showed that SNP1 had moderate polymorphism, whereas SNP2 and SNP3 showed low polymorphism, suggesting that *KRTAP24-1* is relatively conserved in Tibetan sheep. This is consistent with the structural role of KAPs in maintaining fibre integrity. Notably, SNP2–SNP3 and SNP1–SNP3 showed high *D′* values but low *R*^2^ values. This pattern is not contradictory, because *D*′ mainly reflects historical recombination, whereas *R*^2^ is more dependent on allele frequencies and reflects allelic correlation between loci. When allele frequencies differ substantially between loci, *R*^2^ can remain low even when *D′* approaches 1 [32]. Therefore, the low *R*^2^ values are likely a statistical consequence of the marked imbalance between common and rare alleles, rather than evidence of weak linkage disequilibrium. In contrast, both D′ and R^2^ were close to zero for SNP1–SNP2, suggesting that these two loci segregate largely independently in this population. These results indicate that interpretation of linkage relationships among *KRTAP24-1* variants should consider *D′*, *R*^2^, and allele frequencies together, rather than relying on a single LD metric.

### 4.3. Association of Genotypes and Haplotypes with Wool Traits

This study found that *KRTAP24-1* variation was mainly associated with wool length, fibre mechanical properties, and clean fleece yield, rather than showing broad effects on all wool traits. Notably, although the TT genotype of SNP1 (c.191C>T) exhibited greater mean fibre length, it was also associated with lower SFT and SY, indicating that the effect of this locus is not uniformly favourable. SFT is an important wool quality trait that reflects fibre mechanical strength and elasticity and is highly sensitive to internal fibre structure [33]. Studies have shown that KIFs provide skeletal support, whereas the KAP matrix contributes plasticity and stability; together, the cortical composite structure formed by KIFs and KAPs determines fibre extensibility [34]. The transition from the CC to CT genotype may alter the ratio or abundance of KIFs and KAPs, thereby affecting wool extensibility. These findings also suggest a potential biological trade-off among wool length, strength, and yield-related traits. A similar phenomenon has been reported for *KRTAP7-1*, another member of the KRTAP family, in which the AB variant affected both clean fleece yield and staple length [35]. SNP3 (c.656C>T) was significantly associated with LL and CFY, suggesting that this locus may be involved in regulating wool length and clean wool output. However, because the TT genotype of SNP3 occurred at a low frequency and was excluded from the analysis, its effect requires further validation in a larger population. No significant association was detected for SNP2 (c.527G>A), but this does not necessarily indicate that this locus is unrelated to wool traits. The genetic effect of a single locus may be relatively weak [36], and such effects may only become detectable through multilocus or haplotype-based analyses [37]. For example, in the *FTO* and *IGF1* genes, single-locus analyses failed to detect stable associations, whereas haplotype analyses revealed significant associations with obesity-related and growth traits, respectively [38,39].

Haplotype analysis further revealed the combined genetic effects of the three SNPs. Previous studies on the *KRTAP6* family [25,40] and *KRTAP13* family genes [41] have similarly shown that haplotypes composed of multiple variants often explain variation in wool traits better than individual SNPs. This phenomenon supports a genetic model in which complex traits are jointly regulated by multiple loci, suggesting that both single-locus effects and multilocus combination effects should be considered when elucidating the genetic basis of wool traits. In this study, the H1H1 combination showed advantages in wool fibre length and mechanical performance, whereas H2H2 was favourable for clean fleece yield, indicating that different haplotype combinations may correspond to different breeding applications. However, given the limited sample sizes of several diplotype groups (H2H2, *n* = 13), these findings should be regarded as exploratory. Further validation and selective use of these haplotype combinations should be conducted according to specific breeding objectives.

### 4.4. Bioinformatic Analysis of KRTAP24-1

Given that all three SNPs were missense mutations leading to amino acid substitutions, we further investigated their potential functional consequences by performing bioinformatic predictions at both the mRNA and protein levels based on the five haplotypes (H1–H5) actually observed in the study population. Prediction of mRNA secondary structure showed only minor differences in minimum free energy (MFE) among the haplotypes. The wild type haplotype H1 and H2 carrying p.L64P had the same MFE value of −216.30 kcal/mol, whereas H3 (p.G176D), H4 (p.A219V), and H5 (p.L64P + p.G176D) showed slightly increased MFE values by 1.6–1.7 kcal/mol, suggesting a slight reduction in thermodynamic stability. However, increasing evidence indicates that even subtle changes in coding-region mRNA secondary structure can affect translation efficiency and co-translational protein folding by modulating ribosomal elongation rates. Wan et al. [42] demonstrated that RNA secondary structure is extensively coupled with translation, and that structural features within coding regions can regulate elongation rates and promote the sequential folding of nascent peptide domains. Faure et al. [43] further showed that mRNA structure can directly influence protein folding pathways by inducing transient translational pausing. In KRTAP24-1, local conformational changes in mRNA caused by amino acid substitutions may affect ribosome movement, thereby altering the conformation or abundance of the nascent protein and contributing to variation in fiber-related traits.

Protein structure prediction showed that KRTAP24-1 is mainly composed of random coils and exhibits typical features of an intrinsically disordered protein (IDP), which is consistent with the known structural role of KAPs as flexible cross-linking molecules in the wool fiber matrix [44,45]. The p.L64P mutation results in the substitution of leucine by proline. In folded proteins, proline is generally regarded as a helix breaker because its backbone φ dihedral angle is restricted and it lacks an amide hydrogen-bond donor [46]. However, because KRTAP24-1 is dominated by random coils and no obvious α-helical structure was predicted, the potential consequence of p.L64P is unlikely to be disruption of a pre-existing helix. Instead, it may locally restrict backbone conformational flexibility around residue 64. Such local rigidification may alter the conformational dynamics of KRTAP24-1, thereby potentially affecting its transient interactions with keratin intermediate filaments and other KAP members during wool fiber assembly.

The overall GRAVY scores and secondary structure compositions of the five haplotype proteins were largely unchanged, suggesting that the three missense mutations did not substantially alter the global physicochemical properties of KRTAP24-1. Notably, ExPASy ProtParam prediction showed that the instability indices of H2 and H5 were slightly increased, indicating a tendency toward increased instability at the primary-sequence level. In contrast, MutationExplorer-based local energy analysis showed that the Rosetta energies at all three amino acid substitution sites decreased, suggesting a trend toward local energetic optimization. This apparent discrepancy is not contradictory, because the two metrics evaluate different aspects of protein stability. The ProtParam instability index is mainly based on the dipeptide composition of the primary sequence and predicts potential protein instability or proteolytic susceptibility [47], whereas Rosetta energy reflects the local conformational energetics around specific sites in a predicted three-dimensional structural model [48]. Therefore, the two indices are not directly comparable. In addition, the p.G176D and p.A219V substitutions may be accommodated by local side-chain rearrangements after energy minimization, resulting in reduced local Rosetta energies. KyteDoolittle hydrophobicity analysis further indicated that the three substitutions mainly caused local changes in hydrophilicity around the mutation sites, without markedly altering the overall hydrophobic characteristics of the protein. Taken together, these bioinformatic predictions suggest that the three missense mutations in KRTAP24-1 are more likely to exert local rather than global structural effects. Their potential functional consequences may be mediated by changes in local conformational dynamics and intermolecular interactions, rather than by a simple increase or decrease in the overall folding stability of the protein. These interpretations remain computational predictions and require further experimental validation.

The STRING protein–protein interaction network showed that KRTAP24-1 had predicted functional associations with several KAP family members, including KRTAP15-1, KRTAP27-1, KRTAP8-1, KRTAP13-3, KRTAP11-1, and KRTAP3-1. The supporting evidence was mainly derived from text mining, co-expression data, curated databases, and limited experimental evidence. These predicted associations are consistent with the known cooperative roles of KAP family members in wool fiber matrix assembly. Previous experimental studies have demonstrated that KRTAP11-1 can bind to multiple keratins, including K31, K33, and K34, and exhibits self-assembly ability, forming a stable structural network through disulfide bonds [49]. Isothermal titration calorimetry further confirmed that KRTAP8-1 can directly bind to keratin K85, supporting the presence of direct physical interactions between KAPs and keratins [50]. The extent to which amino acid substitutions in KRTAP24-1 may alter its binding affinity with these interaction partners remains to be determined through targeted functional studies, such as co-immunoprecipitation or yeast two-hybrid assays.

This study suggests that *KRTAP24-1* may be a potential candidate gene for the genetic improvement of wool traits in Tibetan sheep, but further validation is still required. Several limitations should be acknowledged. First, although the sample size of 277 Tibetan sheep was sufficient to detect moderate or larger genetic effects, these findings were derived from a single flock, and several diplotype combinations had particularly small sample sizes. Therefore, their generalizability to other Tibetan sheep populations requires validation in independent cohorts. Second, although the bioinformatic predictions of mRNA and protein structures were based on widely accepted algorithms, they should be regarded as preliminary computational hypotheses and need to be experimentally validated using circular dichroism spectroscopy, molecular dynamics simulations, or related approaches. Finally, the effects of these mutations on KRTAP24-1 protein interactions and matrix assembly remain to be directly confirmed through targeted functional experiments.

## 5. Conclusions

This study focused on Tibetan sheep, an indigenous breed adapted to the plateau environment, and, for the first time, characterized the genetic variation, expression profile, and preliminary structural features of the *KRTAP24-1* gene in this breed. The results provide initial evidence that *KRTAP24-1* variation is associated with wool trait variation in Tibetan sheep. Among the three missense variants identified, c.191C>T and c.656C>T showed associations with wool length, strength, and yield related traits, while haplotype-based analysis further supported the contribution of combined *KRTAP24-1* variation to phenotypic differences. The high expression of *KRTAP24-1* in skin is consistent with its potential involvement in wool fibre biology. However, the structural analyses should be interpreted only as preliminary in silico predictions based on the observed haplotypes and do not establish functional causation. Future studies should focus on experimental validation of the functional effects of these mutations and evaluation of the utility of these markers in large-scale breeding populations.

## Figures and Tables

**Table 1:** Nucleotide substitution and alleles of the *KRTAP24-1* gene in the Tibetan sheep.

Title	Substitution	Mutation Type	Amino Acid Change
SNP1	c.191C>T	Missense mutation	p.L64P
SNP2	c.527G>A	Missense mutation	p.G176D
SNP3	c.656C>T	Missense mutation	p.A219V

**Table 2:** Haplotype composition and frequency of the Tibetan sheep *KRTAP24-1* gene SNPs.

Haplotype	SNP1(c.191C>T)	SNP2(c.527G>A)	SNP3(c.656C>T)	Count	Frequency (%)
H1	C	G	C	311	59.13
H2	T	G	C	114	21.67
H3	C	A	C	43	8.17
H4	C	G	T	41	7.79
H5	T	A	C	17	3.23

Note: H1, CGC; H2, TGC; H3, CAC; H4, CGT; H5, TAC.

**Table 3:** Population genetic parameters of three *KRTAP24-1* variants in Tibetan sheep.

SNP	Genotype	Genotype Frequency	Allele	Allele Frequency	Ho	Ne	He	PIC	*p*-HWE
c.191C>T	CC (166)	0.6081	C	0.7491	0.2821	1.602	0.3759	0.3053	<0.001
	CT (77)	0.2821							
	TT (30)	0.1099	T	0.2509					
c.527G>A	GG (209)	0.7770	G	0.8866	0.2193	1.251	0.2011	0.1808	0.1360
	GA (59)	0.2193							
	AA (1)	0.0037	A	0.1134					
c.656C>T	CC (235)	0.8577	C	0.9252	0.1350	1.161	0.1384	0.1289	0.6841
	CT (37)	0.1350							
	TT (2)	0.0073	T	0.0748					

**Table 4:** Association of SNPs with wool traits in Tibetan Sheep.

Traits	SNP1(c.191C>T)			*p* Value	SNP2(c.527G>A)		*p* Value	SNP3(c.656C>T)		*p* Value
(Mean ± SD)	CC (*n* = 166)	CT (*n* = 77)	TT (*n* = 30)		GG (*n* = 209)	GA (*n* = 59)		CC (*n* = 235)	CT (*n* = 37)	
MFL (cm)	**15.95 ± 5.37 ^a^**	**15.47 ± 5.19 ^a^**	**17.74 ± 5.90 ^b^**	**0.041**	15.92 ± 5.50	16.31 ± 5.05	0.420	16.22 ± 5.42	14.74 ± 5.12	0.140
CVFL (%)	24.49 ± 10.60	23.43 ± 7.59	26.74 ± 19.86	0.988	24.06 ± 9.92	25.73 ± 5.13	0.783	24.76 ± 11.83	22.47 ± 6.52	0.542
SL (cm)	10.29 ± 1.62	9.89 ± 1.28	10.26 ± 1.98	0.719	10.25 ± 1.63	9.91 ± 1.77	0.091	10.22 ± 1.68	9.91 ± 1.61	0.606
LL (cm)	15.90 ± 6.42	15.19 ± 6.65	17.48 ± 7.08	0.341	15.78 ± 6.36	16.19 ± 7.30	0.833	**16.02 ± 6.59 ^b^**	**14.97 ± 6.41 ^a^**	**0.034**
MFD (µm)	48.33 ± 7.00	48.61 ± 6.83	48.55 ± 6.64	0.780	48.61 ± 7.10	47.83 ± 6.13	0.574	48.44 ± 6.98	48.38 ± 6.46	0.805
FDSD (µm)	17.57 ± 2.66	17.18 ± 2.63	17.71 ± 2.29	0.797	17.37 ± 2.68	17.84 ± 2.33	0.418	17.50 ± 2.69	17.36 ± 2.07	0.741
CVFD (%)	37.18 ± 8.29	36.07 ± 7.65	37.67 ± 7.61	0.750	36.61 ± 8.27	37.99 ± 7.11	0.479	36.97 ± 8.21	36.59 ± 6.97	0.734
SFBF (cN)	47.70 ± 11.32	45.83 ± 9.59	45.32 ± 8.56	0.161	47.06 ± 10.33	46.43 ± 11.56	0.382	47.05 ± 10.51	46.13 ± 11.22	0.470
EB (%)	41.40 ± 1.02	41.34 ± 1.00	38.60 ± 1.08	0.233	41.30 ± 1.02	40.26 ± 0.99	0.236	41.37 ± 1.00	39.30 ± 1.12	0.212
SFT (cN/mm^2^)	**0.60 ± 0.15 ^B^**	**0.58 ± 0.12 ^B^**	**0.55 ± 0.** **11 ^A^**	**0.007**	0.59 ± 0.13	0.59 ± 0.15	0.464	0.59 ± 0.14	0.58 ± 0.14	0.474
SY (%)	**77.10 ± 0.07 ^b^**	**79.40 ± 0.12 ^b^**	**75.80 ± 0.10 ^a^**	**0.018**	78.80 ± 0.07	77.60 ± 0.09	0.516	77.40 ± 0.08	78.20 ± 0.09	0.741
CFY (%)	48.43 ± 0.13	49.12 ± 0.11	52.23 ± 0.11	0.360	48.32 ± 0.13	51.16 ± 0.10	0.060	**50.10 ± 0.13 ^B^**	**45.30 ± 0.10 ^A^**	**0.002**

Note: Descriptive statistics are presented as mean ± standard deviation. Superscript letters (a, b) indicate group differences based on LMM followed by Tukey–Kramer HSD post hoc tests. For non-normally distributed traits, analyses were performed using RINT data, whereas normally distributed traits were analyzed using untransformed data. Means within the same row with different lowercase superscript letters indicate significant differences, whereas those with different uppercase superscript letters indicate highly significant differences. After FDR correction, *p* < 0.05 was considered significant, whereas *p* < 0.01 was considered highly significant; FDR-adjusted *p*-values meeting these thresholds are shown in bold.

**Table 5:** Association Analysis Between *KRTAP24-1* Haplotype Combinations and Wool Traits.

Traits	Combined Haplotype						*p* Value
(Mean ± SD)	H1H1 (93; 35.40%)	H1H2 (64; 24.30%)	HIH3 (38; 14.40%)	H1H4 (23; 8.70%)	H2H5 (15; 5.70%)	H2H2 (13; 5.00%)	
MFL (cm)	16.46 ± 5.58	15.81 ± 5.19	15.20 ± 4.93	14.86 ± 5.32	18.82 ± 4.84	16.49 ± 6.94	0.369
CVFL (%)	25.38 ± 12.23	23.24 ± 7.66	24.33 ± 8.52	21.47 ± 6.18	30.22 ± 16.18	22.72 ± 7.44	0.925
SL (cm)	10.48 ± 1.61	9.98 ± 1.60	9.94 ± 1.69	10.11 ± 1.65	9.69 ± 2.19	10.92 ± 1.55	0.192
LL (cm)	**16.64 ± 6.42 ^ab^**	**15.04 ± 6.54 ^a^**	**15.07 ± 6.62 ^a^**	**14.73 ± 5.78 ^a^**	**19.84 ± 8.00 ^b^**	**14.76 ± 4.81 ^a^**	**0.024**
MFD (µm)	48.71 ± 7.66	48.70 ± 6.64	47.24 ± 6.08	48.94 ± 5.85	49.71 ± 6.28	47.21 ± 7.05	0.916
FDSD (µm)	17.19 ± 2.92	17.32 ± 2.70	18.38 ± 2.22	17.90 ± 2.18	16.76 ± 2.54	18.80 ± 1.36	0.089
CVFD (%)	36.23 ± 9.23	36.21 ± 7.44	39.57 ± 6.80	37.11 ± 6.33	34.32 ± 7.28	41.54 ± 6.19	0.259
SFBF (cN)	48.49 ± 10.22	45.85 ± 9.86	46.82 ± 13.20	45.91 ± 13.18	44.97 ± 8.12	45.71 ± 9.36	0.082
EB (%)	**42.50 ± 0.93 ^b^**	**41.20 ± 1.04 ^ab^**	**40.90 ± 1.08 ^ab^**	**38.10 ± 1.28 ^a^**	**38.60 ± 0.82 ^a^**	**38.50 ± 1.21 ^a^**	**0.043**
SFT (cN/mm^2^)	0.61 ± 0.13	0.58 ± 0.12	0.59 ± 0.17	0.58 ± 0.16	0.57 ± 0.10	0.57 ± 0.11	0.085
SY (%)	77.80 ± 0.07	79.60 ± 0.07	78.50 ± 0.07	78.90 ± 0.08	73.60 ± 0.12	76.70 ± 0.08	0.349
CFY (%)	**47.20 ± 0.14 ^a^**	**50.00 ± 0.11 ^ab^**	**52.80 ± 0.11 ^ab^**	**46.40 ± 0.11 ^a^**	**49.20 ± 0.10 ^ab^**	**56.90 ± 0.14 ^b^**	**0.011**

Note: Descriptive statistics are presented as mean ± standard deviation. Superscript lowercase letters indicate significant differences among haplotype combination groups based on LMM followed by Tukey–Kramer HSD post hoc tests; values sharing the same letter are not significantly different. Bold values indicate significant associations after FDR correction, with *p* < 0.05 considered significant.

**Table 6:** Analysis of the Physicochemical Properties of Five Haplotypes of KRTAP24-1 Protein.

Haplotype	MW (Da)	pI	Instability Index	Aliphatic Index	GRAVY	Asp + Glu	Arg + Lys
H1	27,876.28	8.84	58.81	51.47	−0.439	10	21
H2	27,860.24	8.84	59.57	49.92	−0.460	10	21
H3	27,934.32	8.77	58.51	51.47	−0.451	11	21
H4	27,904.33	8.84	58.81	52.22	−0.429	10	21
H5	27,918.27	8.77	59.27	49.92	−0.473	11	21

Note: MW, molecular weight; pI, theoretical isoelectric point; GRAVY, grand average of hydropathicity. Asp + Glu indicates negatively charged residues, and Arg + Lys indicates positively charged residues. Proteins with an instability index greater than 40 are predicted to be unstable.

## Data Availability

Data supporting the findings of this study are included in the main text and its Supplementary Materials. Information regarding covariates (age, sex, and sampling batch) used in the association analysis models, genotype calls, the genomic relationship matrix (GRM), results of haplotype and diplotype analyses, and R analysis code are all provided in the Supplementary Files. Raw wool phenotypic data are not publicly available at this time as they are required for the research team’s ongoing work; however, they may be obtained from the corresponding author upon reasonable request.

## Supplementary Materials

The following supporting information can be downloaded at: https://www.mdpi.com/article/10.3390/ani16132111/s1, Table S1: PCR and RT-qPCR primers used for *KRTAP24-1*; Table S2: PCR reaction mixture and amplification program; Table S3: RT-qPCR reaction mixture and amplification program; Table S4: Primers for PARMS genotyping of *KRTAP24-1* SNPs; Supplementary File S1: GRM; Supplementary File S2: Wool Trait Measurement Methods; Supplementary File S3: AF-G3N073-F1 PDB; Supplementary File S4: Genotyping; Supplementary File S5: *KRTAP24-1* haplotype diplotype results; Supplementary File S6: R code; Supplementary File S7: Melting curves; Supplementary File S8: Covariates.

## Abbreviations

The following abbreviations are used in this manuscript:

SNP	Single nucleotide polymorphism
MFL	Mean fiber length
CVFL	Coefficient of variation of fiber
SL	Staple length
LL	Lock length
MFD	Mean fiber diameter
FDSD	Fiber diameter of standard deviation
CVFD	Coefficient of variation of fiber diameter
SFBF	Single fiber breaking force
EB	Elongation at break
SFT	Single fiber tenacity
SY	Scouring yield
CFY	Clean fleece yield
LD	Linkage disequilibrium
