# Supplementary material for: Association of KRTAP24-1 Gene Polymorphisms with Wool Traits in Tibetan Sheep (Ovis aries)
Source: Animals (Basel). 2026 Jul 7;16(13):2111. doi: 10.3390/ani16132111 (PMC13359892; doi:10.3390/ani16132111)
Supplement: Supplementary file 1 [file animals-16-02111-s001.zip › Supplementary File S2(Wool_Trait_Measurement_Methods).pdf]

Wool Trait Measurement Methods and Instruments

(1) **Scoured yield (SY).** Scoured yield (SY) also referred to as washing yield , reflects the proportion of raw wool mass retained after scouring**.**We recorded the raw wool mass (W_0), washed it with a neutral commercial dishwashing detergent (pH 7.5) at 40–45 °C for 5 min (three cycles) to remove grease, sweat, and impurities, rinsed thoroughly with distilled water, dried at 105 °C to constant weight (approximately 4 h), and recorded the scoured mass (W_1). SY was calculated as $SY = W_1 / W_0 \times 100\%$.

(2) **Clean fleece yield (CFY).** Clean fleece yield (CFY) also referred to as sorting yield. It is measured on the scoured wool.We weighed a subsample of the scoured wool (Wf), manually separated coarse wool, fine wool, and heterotypic fibres, removed fine wool, heterotypic fibres, kemp, dead fibres, and residual impurities, and weighed the target wool fibre mass (Wc). CFY was calculated as CFY (%) = Wc / Wf × 100%.

(3) **Mean fibre length (MFL).** We randomly selected 100 single fibres per sample, straightened each fibre against a steel ruler, measured the length individually, and calculated the mean value.

(4) **Coefficient of variation of fibre length (CVF).** CVF was derived from the same 100 fibres as CVF = SD / mean × 100%.

(5) **Mean fibre diameter (MFD), fibre diameter standard deviation (FDSD), and coefficient of variation of fibre diameter (CVFD).** These traits were determined with an automatic fibre fineness tester (BEION F10, Shanghai Beion Scientific Instrument Co., Ltd., Shanghai, China) using at least 5,000 fibre snippets per sample.

(6) **Single fibre breaking force (SFBF), single fibre tenacity (SFT), and elongation at break (EB).** These traits were measured on an electronic single-yarn strength tester (YG020, Changzhou Textile Instrument Factory, Changzhou, China) at a gauge length of 30 mm and a crosshead speed of 10 mm/min, with 50 randomly selected fibres per sample.
