# Supplementary material for: Association of KRTAP24-1 Gene Polymorphisms with Wool Traits in Tibetan Sheep (Ovis aries)
Source: Animals (Basel). 2026 Jul 7;16(13):2111. doi: 10.3390/ani16132111 (PMC13359892; doi:10.3390/ani16132111)
Supplement: Supplementary file 1 [file animals-16-02111-s001.zip › Supplementary Table S1-S4.pdf]

Table S1. PCR and RT-qPCR primers used for *KRTAP24-1*

Primer name	Primer sequence (5'–3')	Annealing temperature (°C)	Segment length (bp)	Application
KRTAP24-1-F1	AAACATCAGCAGCAGCTAAAC	58	869	PCR
KRTAP24-1-R1	GTCTGTAACCACCATACAAATACC			
KRTAP24-1-F2	ATTGCTCTTTGCTCCAGACAT	65	120	RT-qPCR
KRTAP24-1-R2	AGGGCACAGACGAGTTTGATT			
GAPDH-F	CAAGTTCCACGGCACAGTCAA	60	101	Reference gene
GAPDH-R	TGGTCATAAGTCCCTCCACGAT			

Table S2. PCR reaction mixture and amplification program

Category	Item	Condition/Volume
Reaction mixture	Genomic DNA template	0.8 μL
	Forward primer	0.8 μL
	Reverse primer	0.8 μL
	Taq DNA polymerase (2×)	10.0 μL
	ddH_2O	7.6 μL
	Total volume	20.0 μL
PCR program	Initial denaturation	94 °C, 3 min
	Denaturation	94 °C, 30 s
	Annealing	T_a, 30 s
	Extension	72 °C, 30 s
	Cycles	29
	Final extension	72 °C, 2 min
	Hold	4 °C

Table S3. RT-qPCR reaction mixture and amplification program

Category	Item	Condition/Volume
Reaction mixture	TB Green Premix Ex Taq II (2×)	10.0 μL
	Forward primer	0.8 μL
	Reverse primer	0.8 μL
	cDNA template	2.0 μL
	ddH_2O	7.2 μL
	Total volume	20.0 μL
RT-qPCR program	Initial denaturation	95 °C, 4 min
	Denaturation	95 °C, 15 s
	Annealing	65 °C, 30 s
	Extension	72 °C, 30 s
	Cycles	35

Table S4. Primers for PARMS genotyping of *KRTAP24-1* SNPs

Primer name	Primer sequence(5'–3')	Application
KRTAP24-1-1Fc	GAAGGTGACCAAGTTCATGCTCAAGAAACTTGTGGAGAAGCCCC	SNP1 Genotyping
KRTAP24-1-1Ft	GAAGGTCGGAGTCAACGGATTCAAGAAACTTGTGGAGAAGCCCT	
KRTAP24-1-1R	GCAGGTCTTGGGCTCAGAAC	
KRTAP24-1-2Rc	GAAGGTGACCAAGTTCATGCTGAGAAGCCACTGGGTATGAAGC	SNP2 Genotyping
KRTAP24-1-2Rt	GAAGGTCGGAGTCAACGGATTGAGAAGCCACTGGGTATGAAGT	
KRTAP24-1-2F	GCCCCTAAGCCACTACAGACTG	
KRTAP24-1-3Fc	GAAGGTGACCAAGTTCATGCTCCCTCCACTGAGCTGTTTTGC	SNP3 Genotyping
KRTAP24-1-3Ft	GAAGGTCGGAGTCAACGGATTCCCTCCACTGAGCTGTTTTGT	
KRTAP24-1-3R	CTTGGTATAGAGCTTAGAGACCGGAAG	
IC-F	GCCCTGGACTTCGAGCAGGA	Internal Control
IC-R	GCGGCGATGATCTTGATCTTC	
