# Supplementary figures and images for: Association of KRTAP24-1 Gene Polymorphisms with Wool Traits in Tibetan Sheep (Ovis aries)
Source: Animals (Basel). 2026 Jul 7;16(13):2111. doi: 10.3390/ani16132111 (PMC13359892; doi:10.3390/ani16132111)

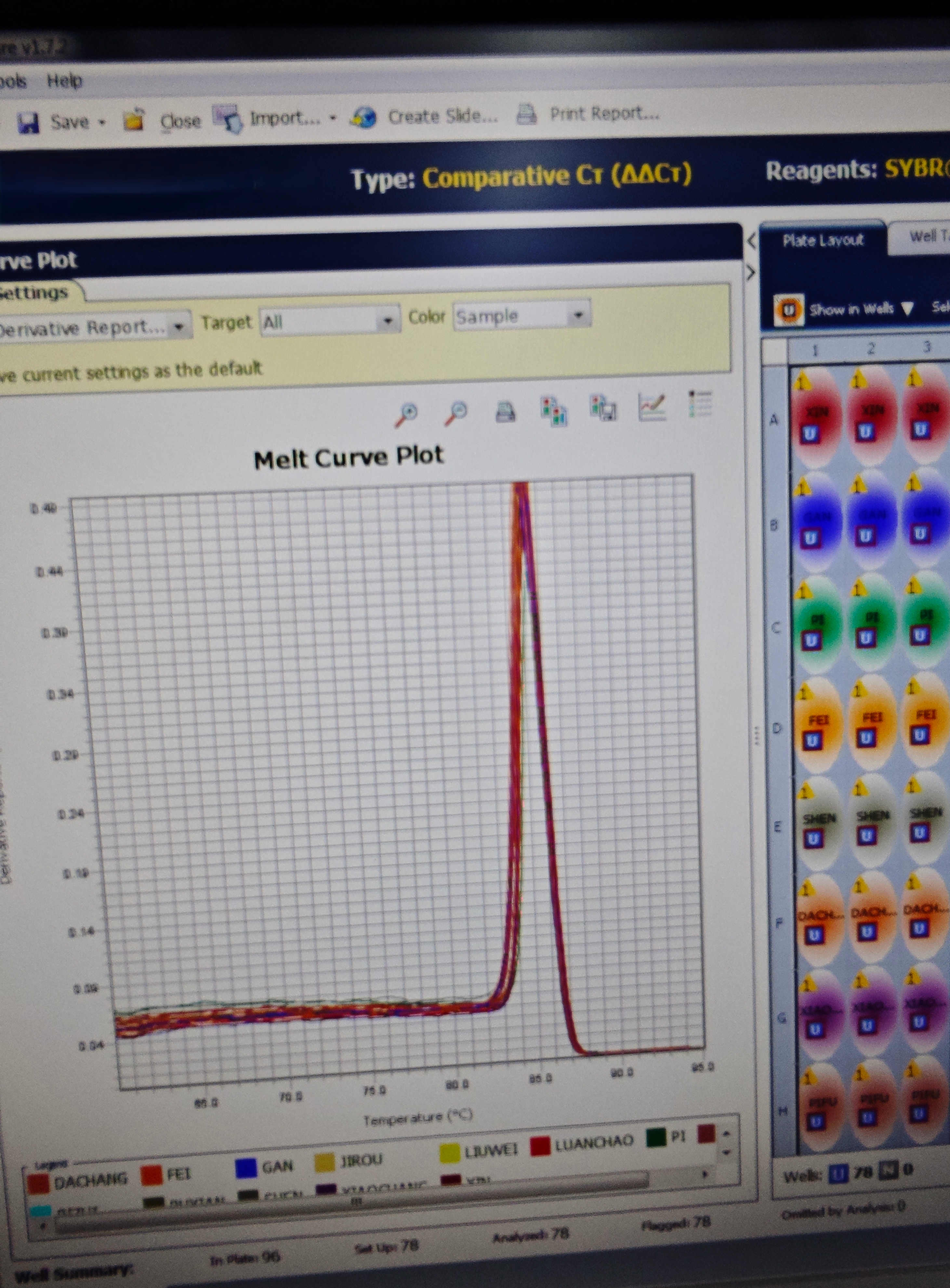

Supplement: Supplementary file 1 [file animals-16-02111-s001.zip › Supplementary File S7(Melting curves )/04452fe5dc457d7ab1f311a19a53ab47.jpg]

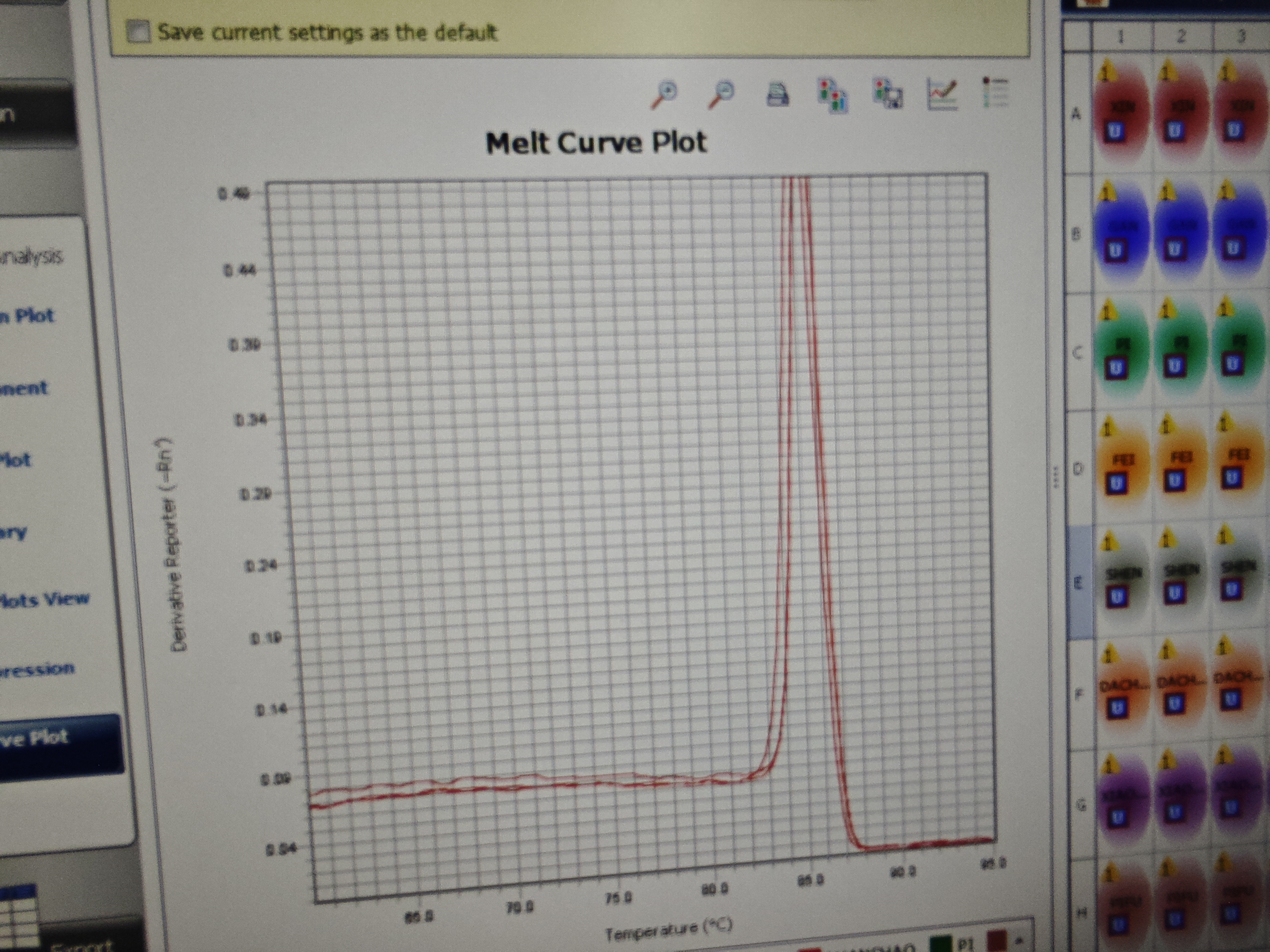

Supplement: Supplementary file 1 [file animals-16-02111-s001.zip › Supplementary File S7(Melting curves )/168b31b4c29bf7cd5afbdfd670680111.jpg]

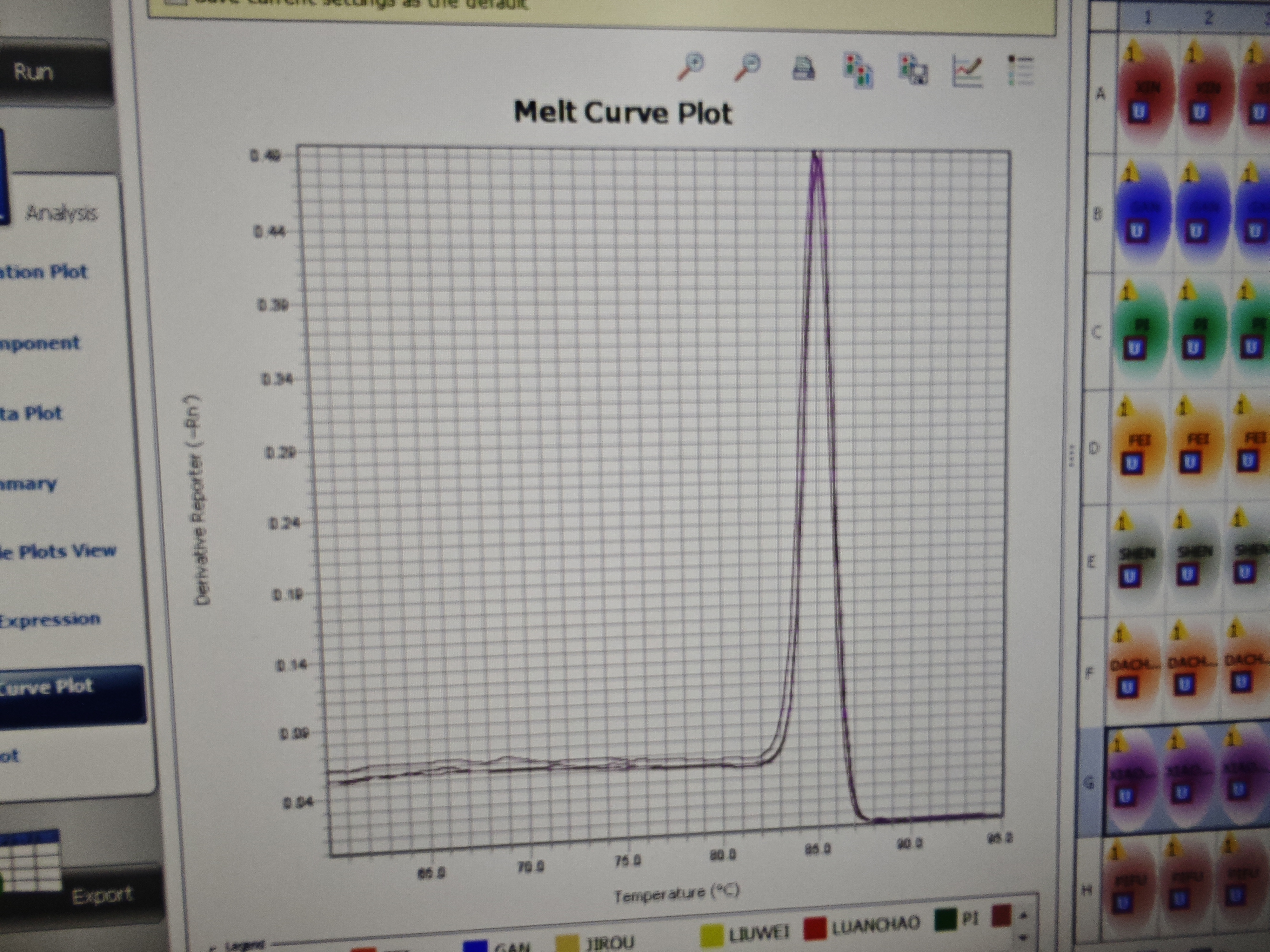

Supplement: Supplementary file 1 [file animals-16-02111-s001.zip › Supplementary File S7(Melting curves )/22b34be0a3ad615e802b990aeb9dff2c.jpg]

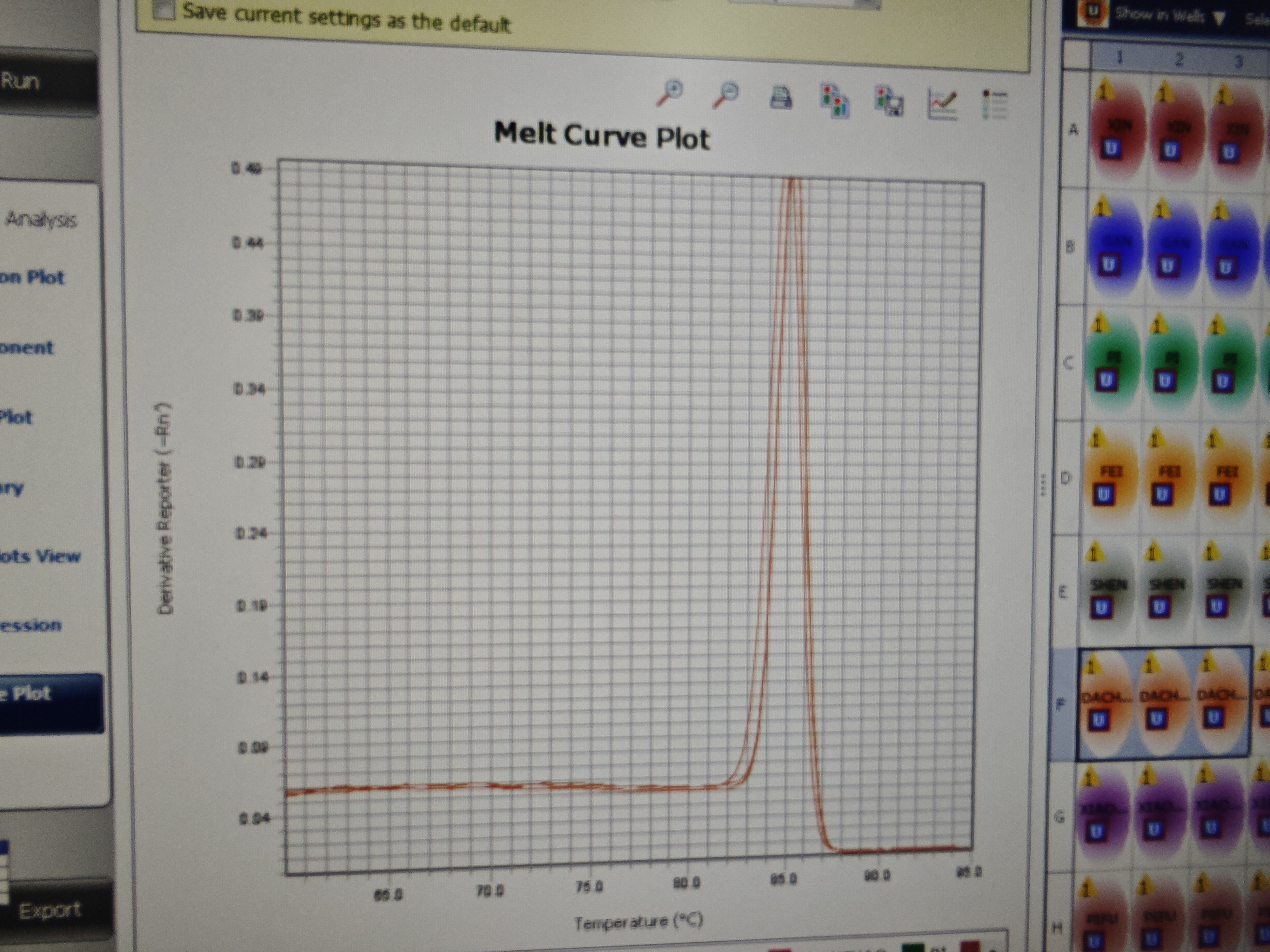

Supplement: Supplementary file 1 [file animals-16-02111-s001.zip › Supplementary File S7(Melting curves )/3065cb9b7c5580bc8e741cd147a65895.jpg]

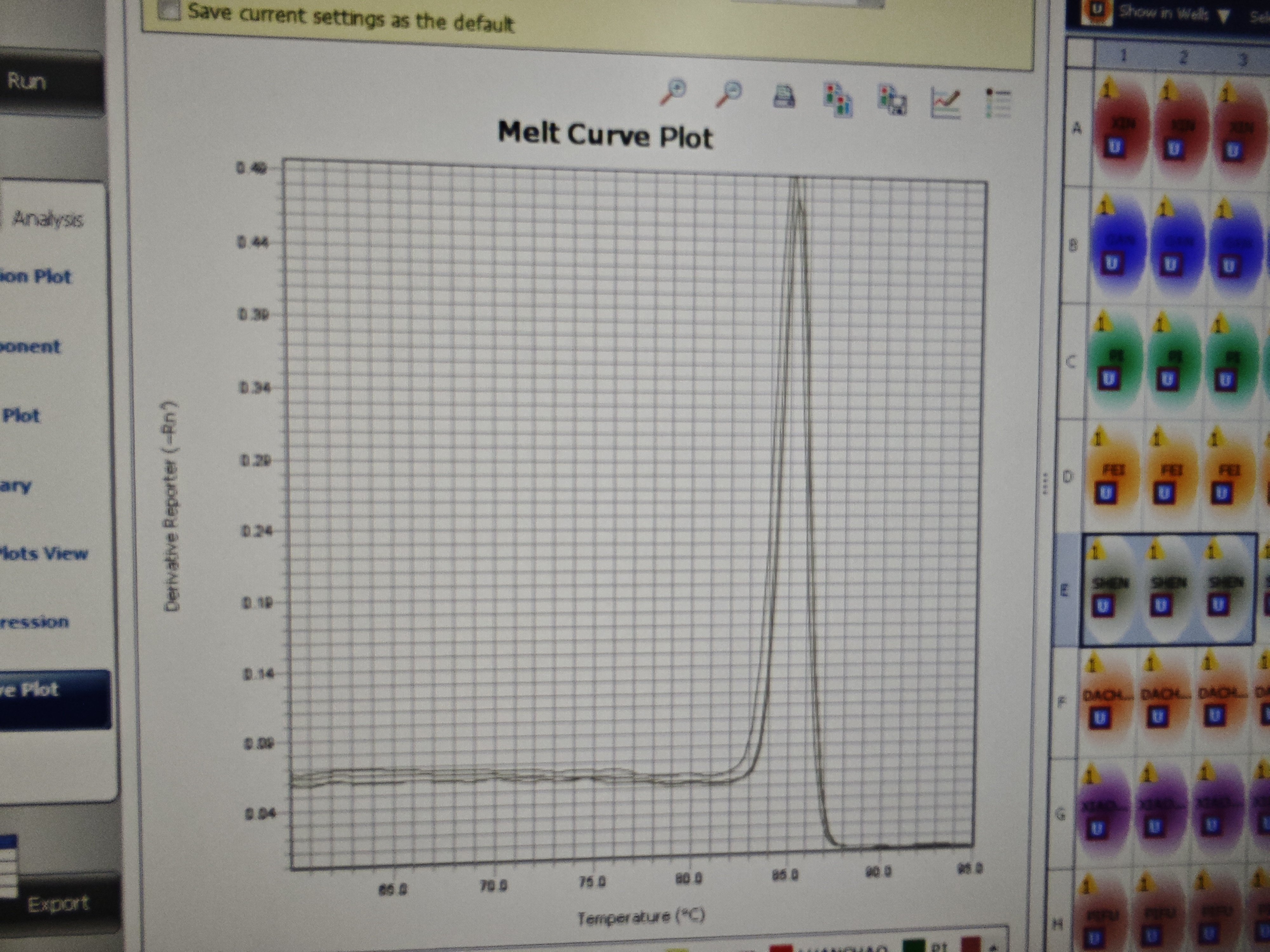

Supplement: Supplementary file 1 [file animals-16-02111-s001.zip › Supplementary File S7(Melting curves )/5702fd6df32411bf585fb96dd976e93d.jpg]

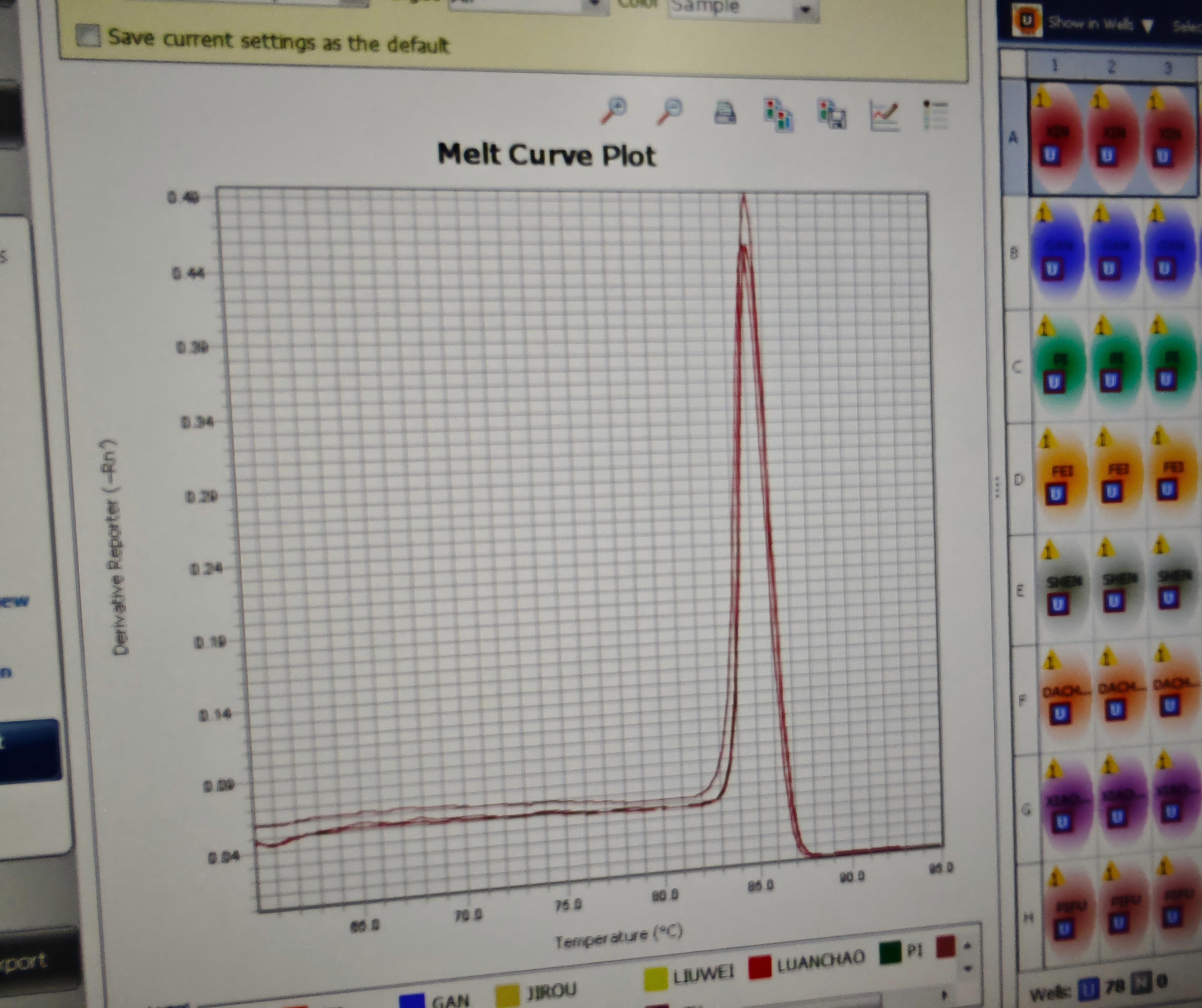

Supplement: Supplementary file 1 [file animals-16-02111-s001.zip › Supplementary File S7(Melting curves )/75fdda4818b218b1e19a782ce8cc3e30.jpg]

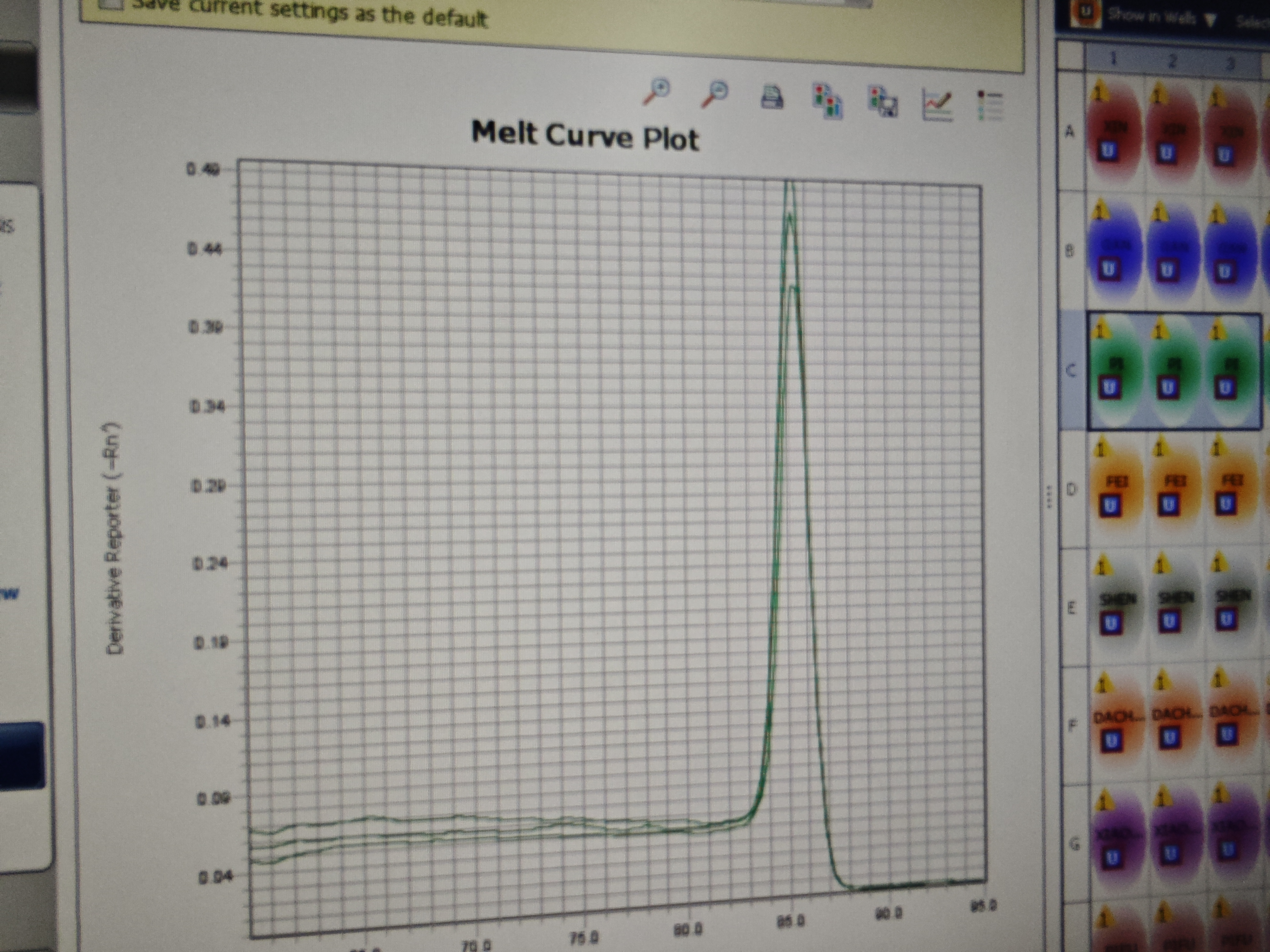

Supplement: Supplementary file 1 [file animals-16-02111-s001.zip › Supplementary File S7(Melting curves )/774546e2aa08f82453fb30903c05357c.jpg]

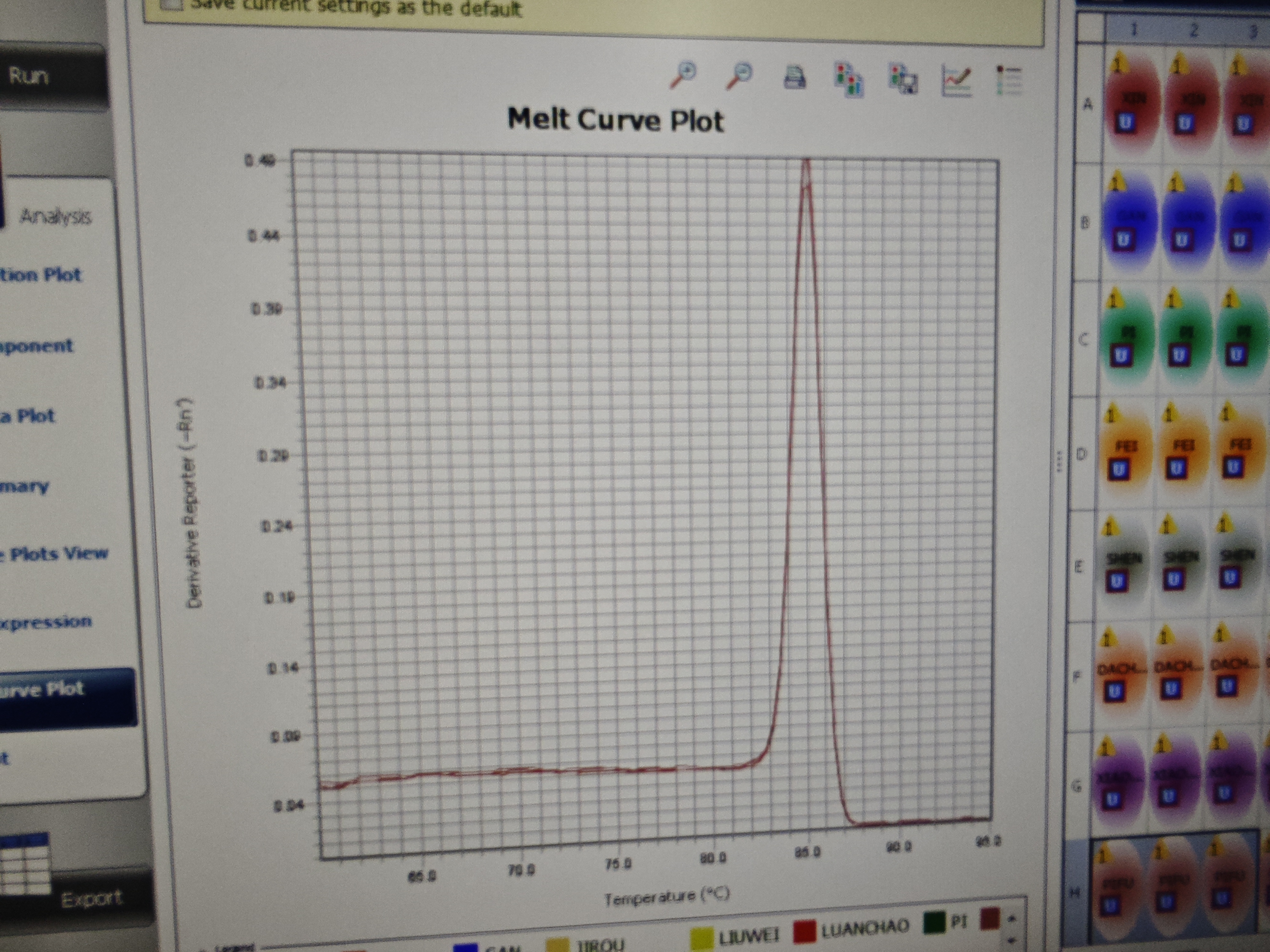

Supplement: Supplementary file 1 [file animals-16-02111-s001.zip › Supplementary File S7(Melting curves )/77fdf4d0c5bb76e5a4d6974f12209bb8.jpg]

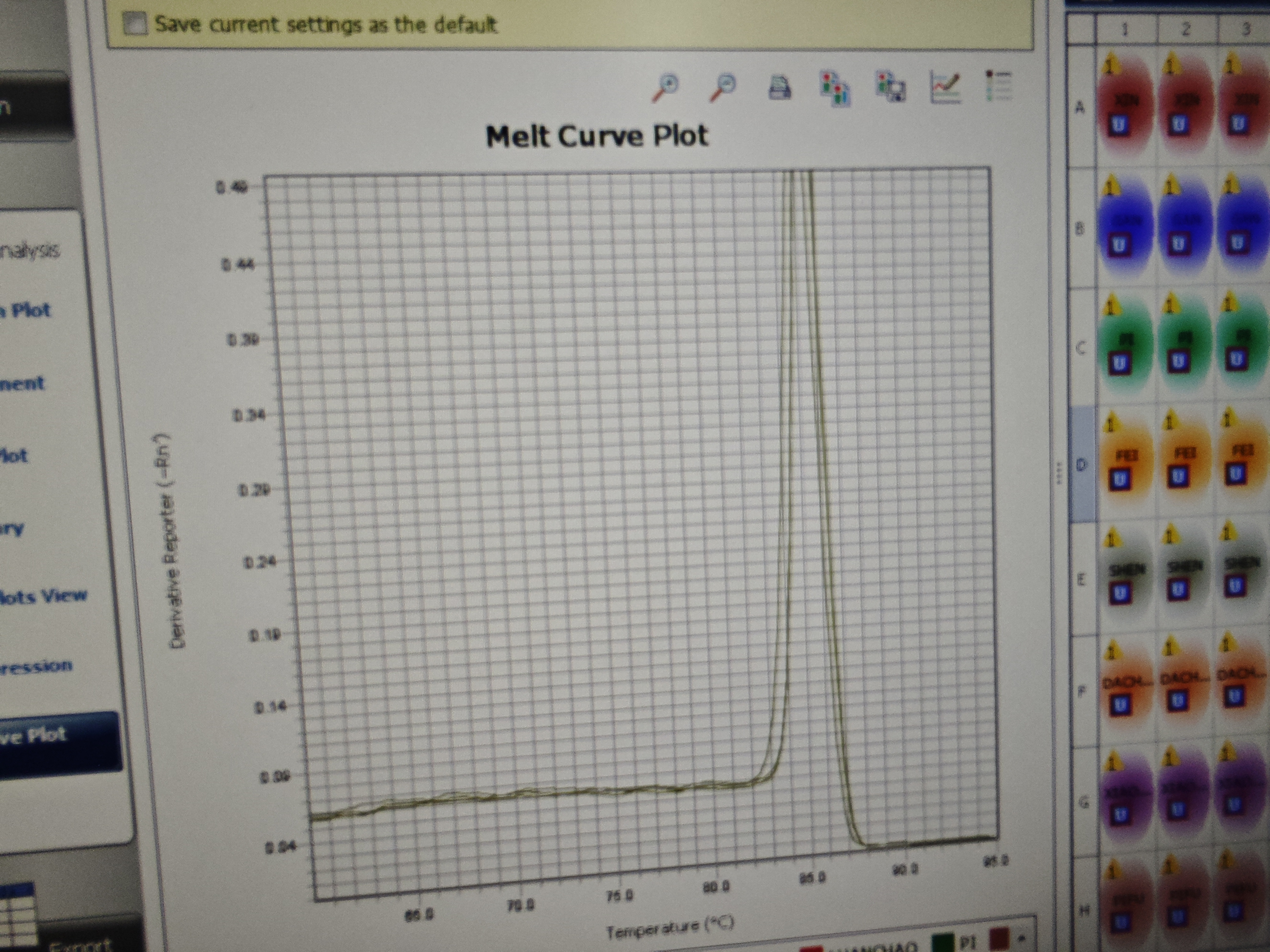

Supplement: Supplementary file 1 [file animals-16-02111-s001.zip › Supplementary File S7(Melting curves )/8630875fbbcfa90e87d2fdd262f0a8b2.jpg]

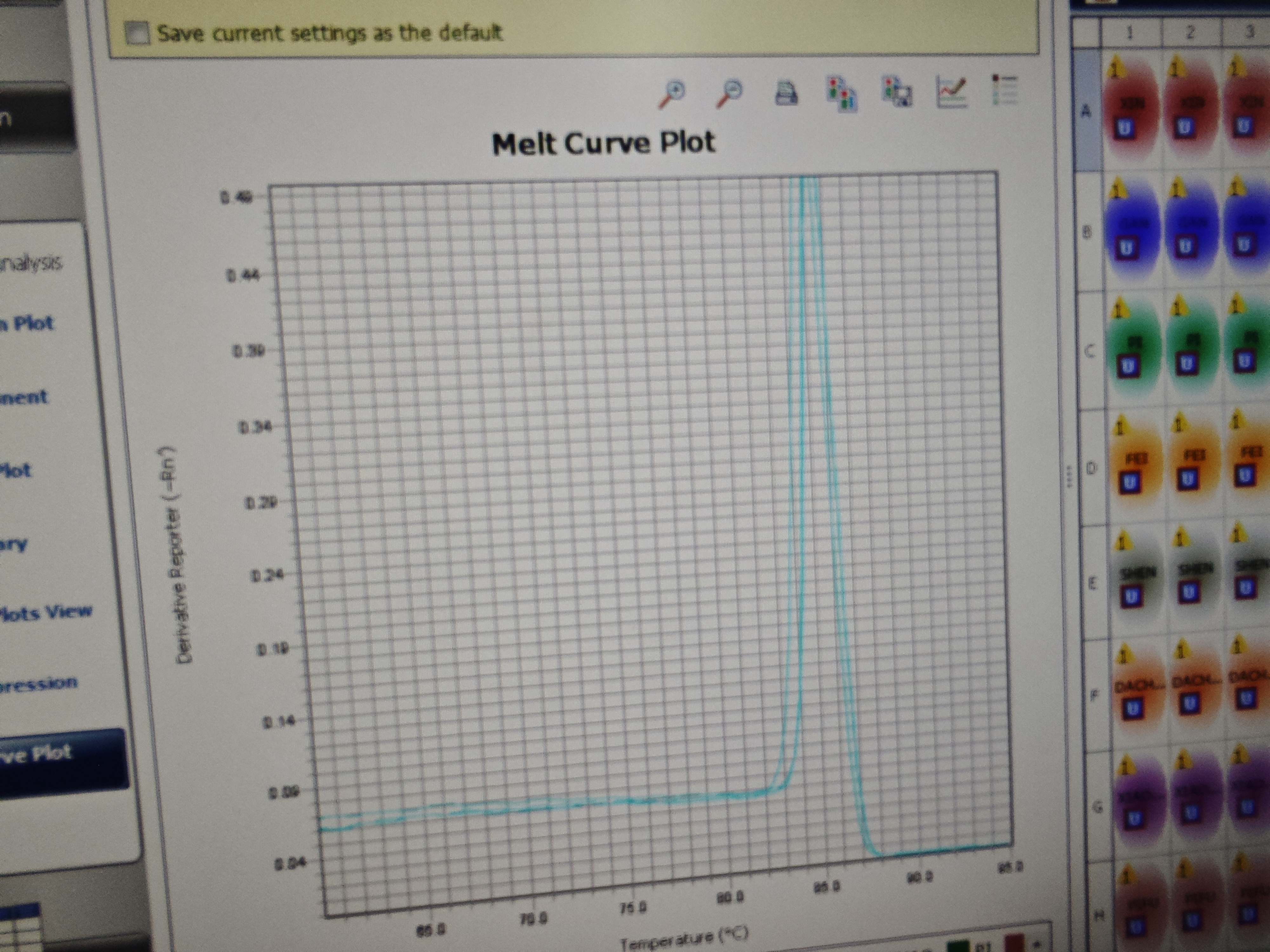

Supplement: Supplementary file 1 [file animals-16-02111-s001.zip › Supplementary File S7(Melting curves )/96af8e033a69a571d4284e07afae0644.jpg]

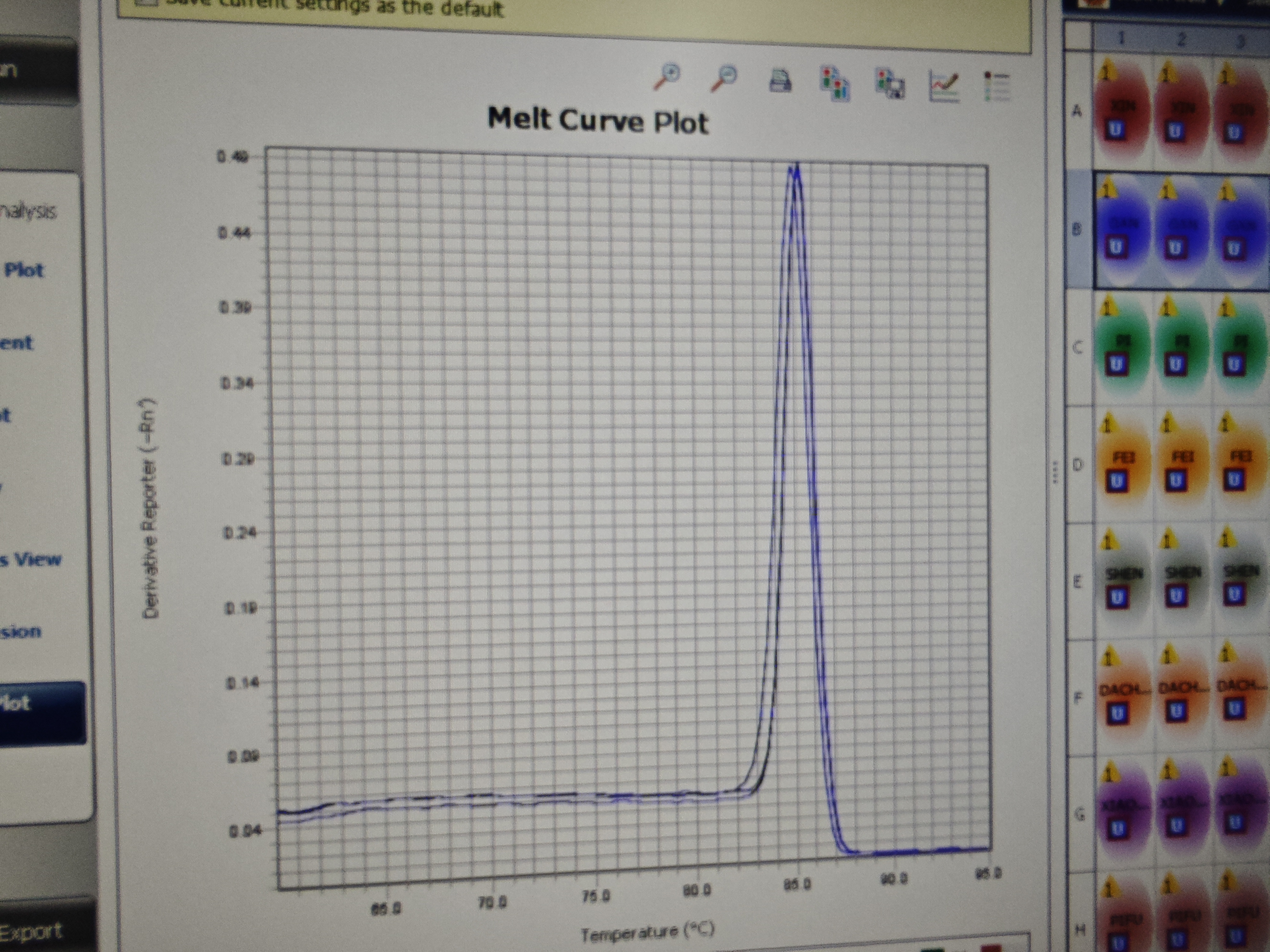

Supplement: Supplementary file 1 [file animals-16-02111-s001.zip › Supplementary File S7(Melting curves )/db5d62f784cfb98cc7f450e0e11ba958.jpg]

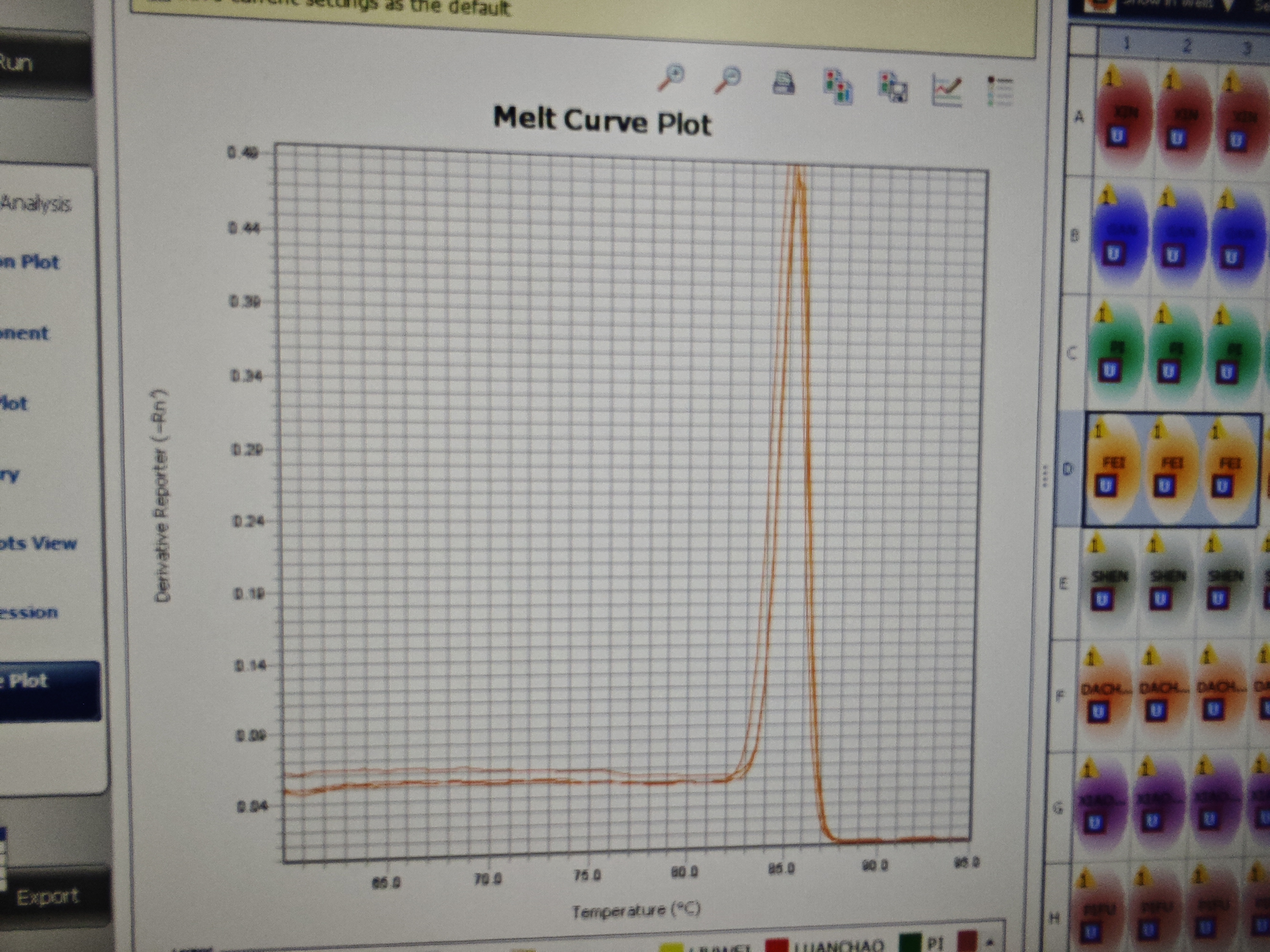

Supplement: Supplementary file 1 [file animals-16-02111-s001.zip › Supplementary File S7(Melting curves )/e36a2cb2413b7b49a6c73fe0b1b37870.jpg]

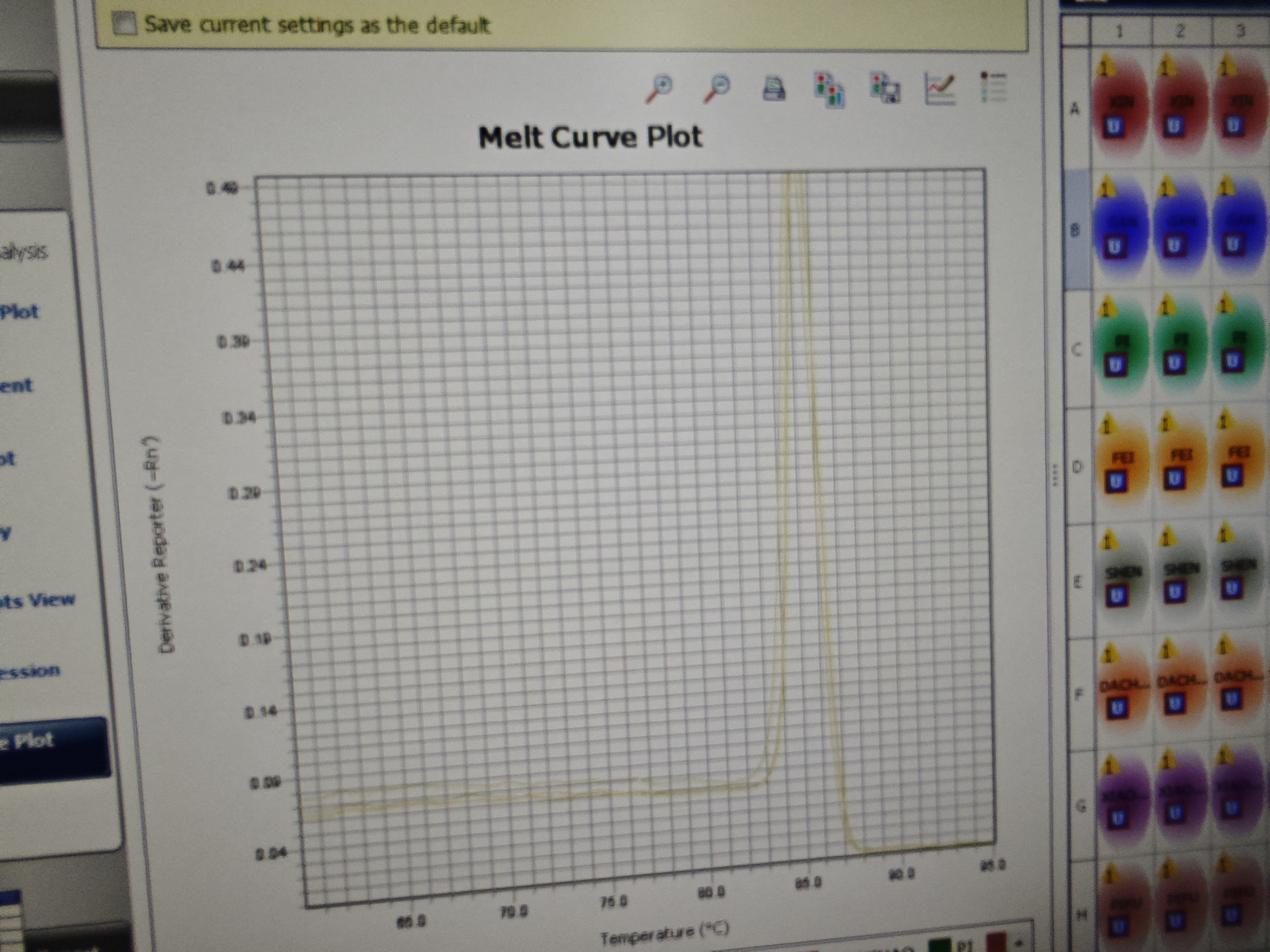

Supplement: Supplementary file 1 [file animals-16-02111-s001.zip › Supplementary File S7(Melting curves )/ff5be17ed35770e9aae4351dbb2238aa.jpg]

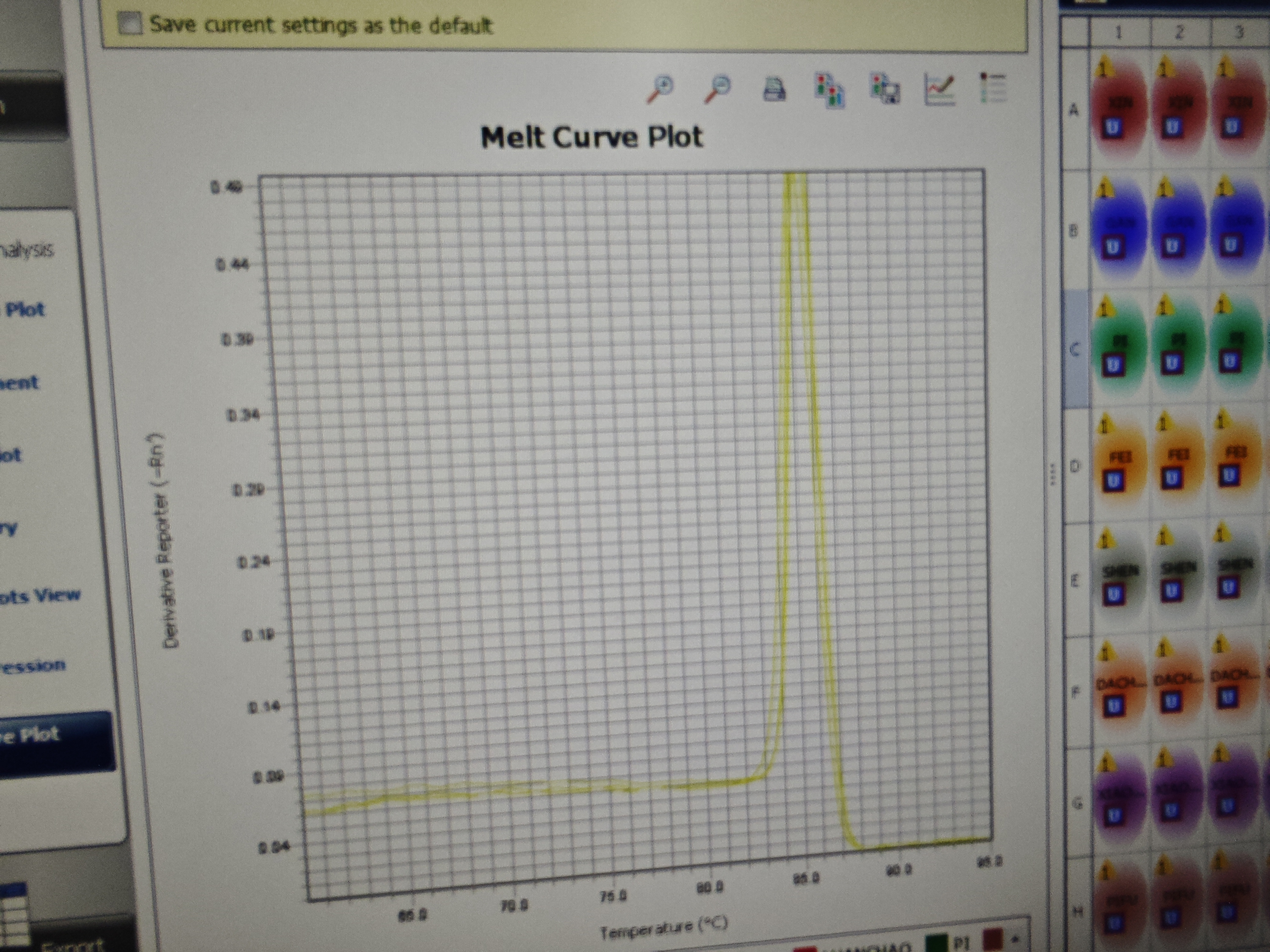

Supplement: Supplementary file 1 [file animals-16-02111-s001.zip › Supplementary File S7(Melting curves )/ff5c0ac53da4dc397e579809935ee07c.jpg]
